# Cell cycle stage-specific transcriptional activation of cyclins mediated by HAT2-dependent H4K10 acetylation of promoters in *Leishmania donovani*

**DOI:** 10.1371/journal.ppat.1006615

**Published:** 2017-09-22

**Authors:** Udita Chandra, Aarti Yadav, Devanand Kumar, Swati Saha

**Affiliations:** Department of Microbiology, University of Delhi South Campus, New Delhi, India; University of York, UNITED KINGDOM

## Abstract

Chromatin modifications affect several processes. In investigating the *Leishmania donovani* histone acetyltransferase HAT2, using *in vitro* biochemical assays and HAT2-heterozygous genomic knockout we found the constitutively nuclear HAT2 acetylated histone H4K10 *in vitro* and *in vivo*. HAT2 was essential. HAT2-depleted cells displayed growth and cell cycle defects, and poor survival in host cells. Real time PCR and DNA microarray analyses, as well as rescue experiments, revealed that downregulation of cyclins CYC4 and CYC9 were responsible for S phase and G2/M defects of HAT2-depleted cells respectively. *Leishmania* genes are arranged in unidirectional clusters, and clustered genes are coordinately transcribed as long polycistronic units, typically from divergent strand switch regions (dSSRs) which initiate transcription bidirectionally on opposite strands. In investigating the mechanism by which CYC4 and CYC9 expression levels are reduced in HAT2-depleted cells without other genes in their polycistronic transcription units being coordinately downregulated, we found using reporter assays that CYC4 and CYC9 have their own specific promoters. Chromatin immunoprecipitation assays with H4acetylK10 antibodies and real time PCR analyses of RNA suggested these gene-specific promoters were activated in cell cycle-dependent manner. Nuclear run-on analyses confirmed that CYC4 and CYC9 were transcriptionally activated from their own promoters at specific cell cycle stages. Thus, there are two tiers of gene regulation. Transcription of polycistronic units primarily initiates at dSSRs, and this most likely occurs constitutively. A subset of genes have their own promoters, at least some of which are activated in a cell-cycle dependent manner. This second tier of regulation is more sensitive to H4K10 acetylation levels, resulting in downregulation of expression in HAT2-depleted cells. This report presents the first data pointing to cell cycle-specific activation of promoters in trypanosomatids, thus uncovering new facets of gene regulation in this parasite family.

## Author summary

*Leishmania donovani* causes Visceral Leishmaniasis (VL), a disease that plagues the world’s poor, particularly in Brazil, Sudan and the Indian sub-continent. If not treated in timely manner VL can be fatal, and due to emerging drug resistance the search for new drug targets continues. Thus, the parasite’s cellular processes remain an area of continued research interest. Eukaryotic DNA is packaged into nucleosomes to compact it, and the histone proteins that constitute a major part of the nucleosomes carry several modifications that affect biological processes. Our laboratory has been studying histone modifications with special attention to the impact of histone acetylation events in the parasite. The present report addresses the significance of one particular histone acetylation event, H4K10, and the protein that mediates this acetylation, HAT2. We find that HAT2 is essential for cell survival, and decrease in HAT2 levels leads to growth and cell cycle defects. From the results of further experiments performed by us we attribute these defects to lowered expression of certain cyclins. We find that the expression of these cyclins are activated in a cell cycle-dependent manner, and this activation is regulated by H4K10 acetylation.

## Introduction

Histone post-translational modifications (PTMs) impact nuclear processes either by generally altering chromatin structure to make it more permissive/repressive to incoming transcription/replication/repair machinery, or by serving as flags recognized by specific proteins that modulate the various DNA-related processes. Due to the wide range of cellular processes they regulate the functional roles of histone PTMs have been extensively investigated. Though these are highly conserved among most eukaryotes, trypanosomatid histones are divergent in sequence [1–3]. Trypanosomatids include several protozoan pathogens that cause communicable diseases such as sleeping sickness, Chagas disease and various forms of Leishmaniases. Visceral Leishmaniasis (VL), caused by *Leishmania donovani* in the Indian subcontinent, can be fatal if not treated at the appropriate time. Due to the emergence of drug resistant strains coupled to risks of dual HIV-*Leishmania* infection the study of the organism’s cellular biology remains an area of continued interest. While histone PTMs have not been globally identified in any *Leishmania* species so far, they are likely to carry the same repertoire of modifications as *Trypanosoma* species since the histone sequences are highly conserved between these family members [4].

Histone acetylation and deacetylation events are dynamic in nature and their timing is regulated by the cellular milieu. Targeting lysine residues (and arginine residues to a lesser degree) in either the N-terminal tails of histones or in the histone core, histone acetylations modulate a myriad of processes such as histone deposition, transcription, DNA replication, and DNA repair, primarily by loosening the interaction between the histone and DNA in the nucleosome, thus decreasing nucleosomal stability (reviewed in [5]). In *Trypanosoma* species, histone acetylations have been identified at H4K2, H4K4, H4K10, H4K14, H3K76, H3K23 and H3K19, and most unusually, at multiple residues on the H2B C-terminal tail [1–3,6]. The enzymes that mediate histone acetylation (histone acetyltransferases or HATs) fall into two categories–those that acetylate histones prior to nucleosomal deposition (Type B HATs), and those that typically acetylate nucleosomal histones (Type A HATs). Four MYST-family (Type A) HATs were annotated in the *Leishmania* whole genome sequences [7,8]. *Leishmania donovani* HAT3 has been found to acetylate H4K4 *in vitro* as well as *in vivo*, and deletion of LdHAT3 leads to deficiencies in histone deposition. LdHAT3 has been identified as an essential player in UV-induced DNA damage repair pathway, via its mediation of PCNA acetylation that must precede PCNA monoubiquitination in order for translesion DNA synthesis-based repair to occur efficiently [9]. *Leishmania donovani* HAT4 acetylates H4K14 *in vitro*, and its deletion leads to prolonged G2/M phase due to down regulation of Cdc20 [10]. *Trypanosoma brucei* HAT1 and HAT2 have been found to be essential, with TbHAT1 knockdown compromising DNA replication as well as telomeric silencing [11]. TbHAT3, though non-essential [11], has been found to facilitate DNA repair at internal sites within chromosomes by promoting Rad51-dependent homologous recombination [12]. In investigating the role of the *Leishmania donovani* MYST-family histone acetyltransferase HAT2, the present study uncovers new facets of gene regulation in this parasite.

## Results

### The constitutively nuclear LdHAT2 acetylates H4K10 *in vitro* and *in vivo*

**Fig 1:**
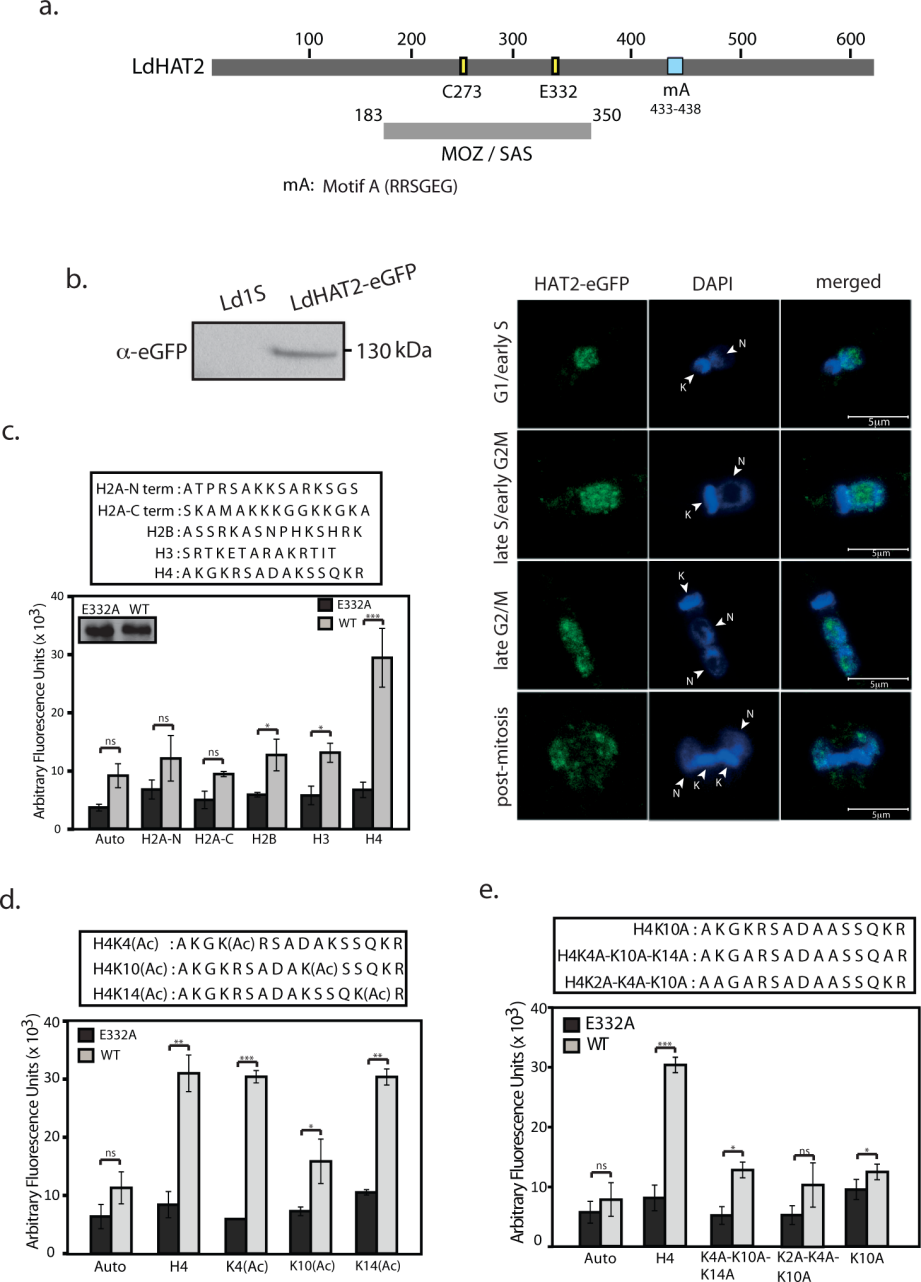
HAT2 is constitutively nuclear and acetylates H4K10 *in vitro*. **a.** Schematic depiction of conserved motifs and residues in the *Leishmania donovani* HAT2 gene. MOZ/SAS: conserved core catalytic acetyltransferase domain. Cys273 and Glu332: the conserved catalytically important cysteine and glutamate residues. **b.** Left panel: Western blot analysis of immunoprecipitation reaction from transfectant cells expressing the tagged HAT2-eGFP, performed using anti-eGFP antibodies (1:2000 dilution; 6.75x10^8^ cells used per IP reaction). Right panels: IFA of HAT2-eGFP at different cell cycle stages. DAPI: stains DNA compartments; N: nucleus, K: kinetoplast. G1/early S: one nucleus, one short kinetoplast (1N1K); late S/early G2/M: one nucleus, one elongated kinetoplast (1N1K); late G2/M: two nucleii, one kinetoplast (2N1K); post-mitosis—two nucleii, two kinetoplasts (2N2K). Imaging done using a LeicaTCS SP5 confocal microscope, 100X (in oil) objective; analysis using Leica LAS AF software. Magnification bar: 5 μm. **c.—e.** HAT assays performed with various peptide substrates (sequences in boxes above graphs). Auto: no peptide added. Inset (first panel): western blot analysis of 1/6 pulled-down fraction.

The cloned *Leishmania donovani* 1S HAT2 (S1 Methods; GenBank Accession No. KY445835) was found to carry the core catalytic acetyltransferase domain that is typical of the members of the MYST family (Fig 1A, MOZ/SAS box). The MOZ/SAS box harboured the conserved cysteine and glutamate residues (Cys273 and Glu332) that have been identified as critical to catalysis—catalysis occurs via a ping pong mechanism wherein the glutamate residue arbitrates the deprotonation of the cysteine residue, which then catalyzes the transfer of the acetyl group from acetyl-CoA to the target lysine residue on the histone substrate via an acetyl-Cys catalytic intermediate [13]. By tagging one of the genomic alleles with eGFP (S1 Methods; S1A Fig), followed by immunofluorescence analysis of the tagged protein at different cell cycle stages using kinetoplast morphology and segregation pattern as marker [14], the protein was found to be constitutively nuclear like LdHAT3 [9] (Fig 1B, S1B Fig). *In vitro* biochemical assays were performed using peptide substrates whose sequences were derived from the termini of *Leishmania* histones. For this purpose the HAT2 proteins (wild type and mutant E332A) were episomally expressed in fusion with FLAG tag in *Leishmania* promastigotes (S1 Methods; S2A Fig), and the FLAG-tagged HAT2 proteins were pulled down and HAT assay performed as described in Materials and Methods. LdHAT2-FLAG had autoacetylation activity, in addition to targeting the N-terminus of histone H4 (Fig 1C). The LdHAT2-E332A-FLAG was catalytically inactive as predicted. Using H4 peptide substrates that were pre-acetylated at K4/K10/K14 as well as various H4-derived peptides where specific lysine residues had been mutated to alanine residues, it was revealed that H4K10 was the primary target of LdHAT2-mediated histone acetylation *in vitro* (Fig 1D and 1E). These findings are in keeping with results from TbHAT2 [11]. To determine if H4K10 is the target of LdHAT2-mediated acetylation *in vivo* also, we raised H4acetylK10-specific antibodies (S1 Methods) and confirmed their specificity using peptide competition assays [9](S2B Fig). H4K10 acetylation was maintained at more or less equivalent levels at all stages of *Leishmania* (S2C Fig), and was detected only in the nucleus at all stages of cell cycle (S2D Fig). Coupled to previous data from the lab indicating rapid nuclear import of histones post-synthesis [9], it appears that acetylation most likely takes place in the nucleus, in keeping with the fact that LdHAT2 is constitutively nuclear.

**Fig 2:**
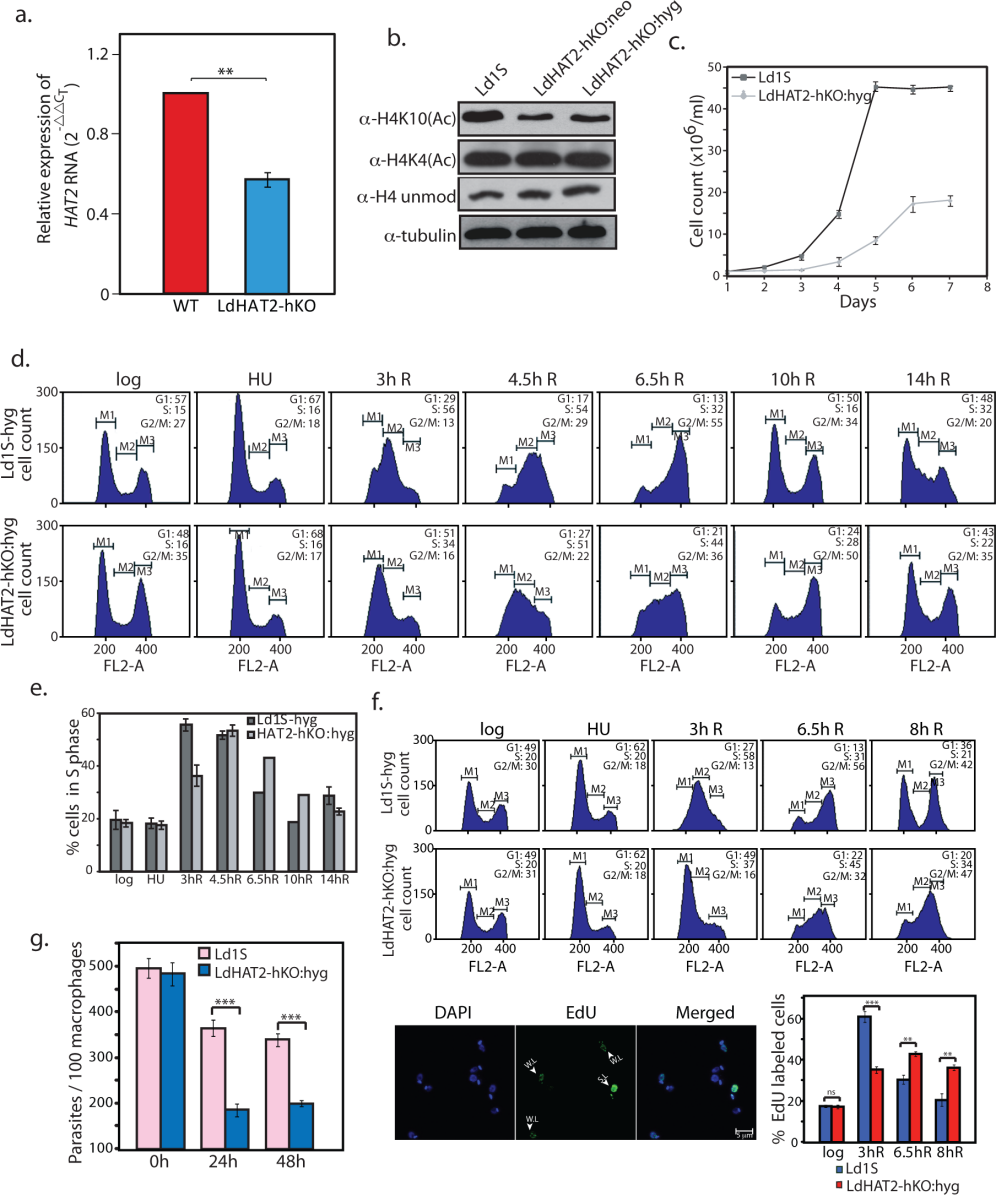
HAT2 acetylates H4K10 *in vivo* and its depletion causes growth and cell cycle defects. **a.** Analysis of differential expression of LdHAT2 in LdHAT2-hKO versus wild type (WT) cells, by real time PCR analysis using 2^-ΔΔC^_T_ method. Internal control: tubulin. Data is average of three experiments, error bars represent standard deviation. Two-tailed student’s t-test was applied: **p < 0.005 **b.** Western blot analysis of whole cell lysates of logarithmically growing wild type and LdHAT2-hKO promastigotes (3.5x10^6^ cell equivalents per lane). **c.** Growth analyses of LdHAT2-hKO cells in comparison with control. Three experiments were initiated in parallel. Data is the average of three experiments, error bars represent standard deviation. **d.** Comparative flow cytometry analysis of HU-synchronized LdHAT2-hKO and control cells: cells blocked at G1/S, then released into S phase. Time after release at which sampling was done is indicated above each column of histograms. 30,000 events analyzed at every time-point; M1, M2 and M3 gates indicate G1, S and G2/M phases. Percent of cells at different stages indicated in histogram insets. Experiment was performed three times; one data set shown here. **e.** Bar graph compares number of cells in S phase at different time-points. Average of three experiments is plotted, error bars depict standard deviation. **f.** HU-synchronized promastigotes were released into S phase, and aliquots of cells pulsed with EdU 3 hours, 6.5 hours and 8 hours after release (15 min pulses). Experiment was performed thrice. Upper panels: flow cytometry profiles of the cells at different intervals. Lower left panels: representative microscopy field with EdU-labelled and unlabeled cells. SL: strongly labeled. WL: weakly labeled. Lower right panel: bar chart comparing percent EdU-labeled cells at each time-point. ~ 100–120 cells were analyzed at each time-point. Data is average of three experiments, error bars—standard deviation. Two-tailed student’s t-test was applied: ns non-significant; **p < 0.005; ***p < 0.0005. **g.** Analysis of LdHAT2-hKO parasites within macrophages (*Leishmania* cycles between insect and mammalian host, existing in the mid-gut of insect as non-infective procyclic promastigotes and later in the salivary glands as infective metacyclic promastigotes, before being released into the mammalian host’s bloodstream via insect bite where they take up residence in macrophages and continue to multiply asexually). Parasites were scored by DAPI-staining of infected cells followed by Z-stack imaging using confocal microscopy. Three experiments were initiated in parallel. Bar chart represents average values of three experiments with error bars indicating standard deviation. Two-tailed student’s t-test was applied: ***p < 0.0005.

As *Leishmania donovani* lacks a canonical RNAi pathway, RNAi-mediated knockdown of genes cannot be carried out. Hence we attempted to create HAT2 genomic knockout. Heterozygous knockouts LdHAT2-hKO:neo and LdHAT2-hKO:hyg were created (S1 Methods) and their authenticity verified (S3A and S3B Fig). However, repeated efforts to obtain HAT2-nulls failed, suggesting that LdHAT2 is essential to cell survival. To confirm this, LdHAT2 was ectopically expressed in LdHAT2-hKO:hyg cells first, and cells of the resultant LdHAT2-hKO:hyg/HAT2-eGFP-blecherry line (S1 Methods) were then transfected to replace the second genomic allele of HAT2. In this case, a successful replacement could be obtained (S3C Fig). The importance of LdHAT2 in mediating cell survival was in contrast to LdHAT3 and LdHAT4, both of which are dispensable [9,10]. Expression of HAT2 in LdHAT2-hKO:hyg cells was ~0.6 of that seen in wild type cells (Fig 2A), and western blot analysis revealed that H4K10 acetylation was reduced by ~ 50% in LdHAT2-hKO cells while H4K4 acetylation levels remained unaltered (Fig 2B). These data confirmed that LdHAT2 mediates H4 acetylation at K10 residue *in vivo* also.

### HAT2 depletion results in aberrant cell cycle patterns and poor propagation within host cells

All further analyses were carried out using the HAT2 heterozygous knockout LdHAT2-hko:hyg. When the effect of HAT2 depletion on cell growth was monitored over a period of seven days it was observed that LdHAT2-hKO:hyg cell cultures grew considerably slower than control Ld1S-hyg cells (Ld1S carrying hygromycin resistance cassette;[9]), never reaching the same cell density as the control (Fig 2C). On scoring the percent of survivors every 24 hours over the seven-day period we found that the difference was not significant enough to account for the disparity in growth rate (S3D Fig). The slow growth of HAT2-depleted cells was subsequently determined to be due to increase in generation time to ~18.6 hours (compared to ~ 9.4 hours in case of control cells; S1 Methods and S3E Fig). Work from our laboratory has found H4K4 acetylation to play a role in histone deposition in *Leishmania* [9]. However, analysis of the DNA-associated protein fraction of LdHAT2-hKO cells in comparison with Ld1S-hyg cells revealed that H4K10 acetylation does not impact nucleosomal deposition (S1 Methods and S3F Fig).

**Fig 3:**
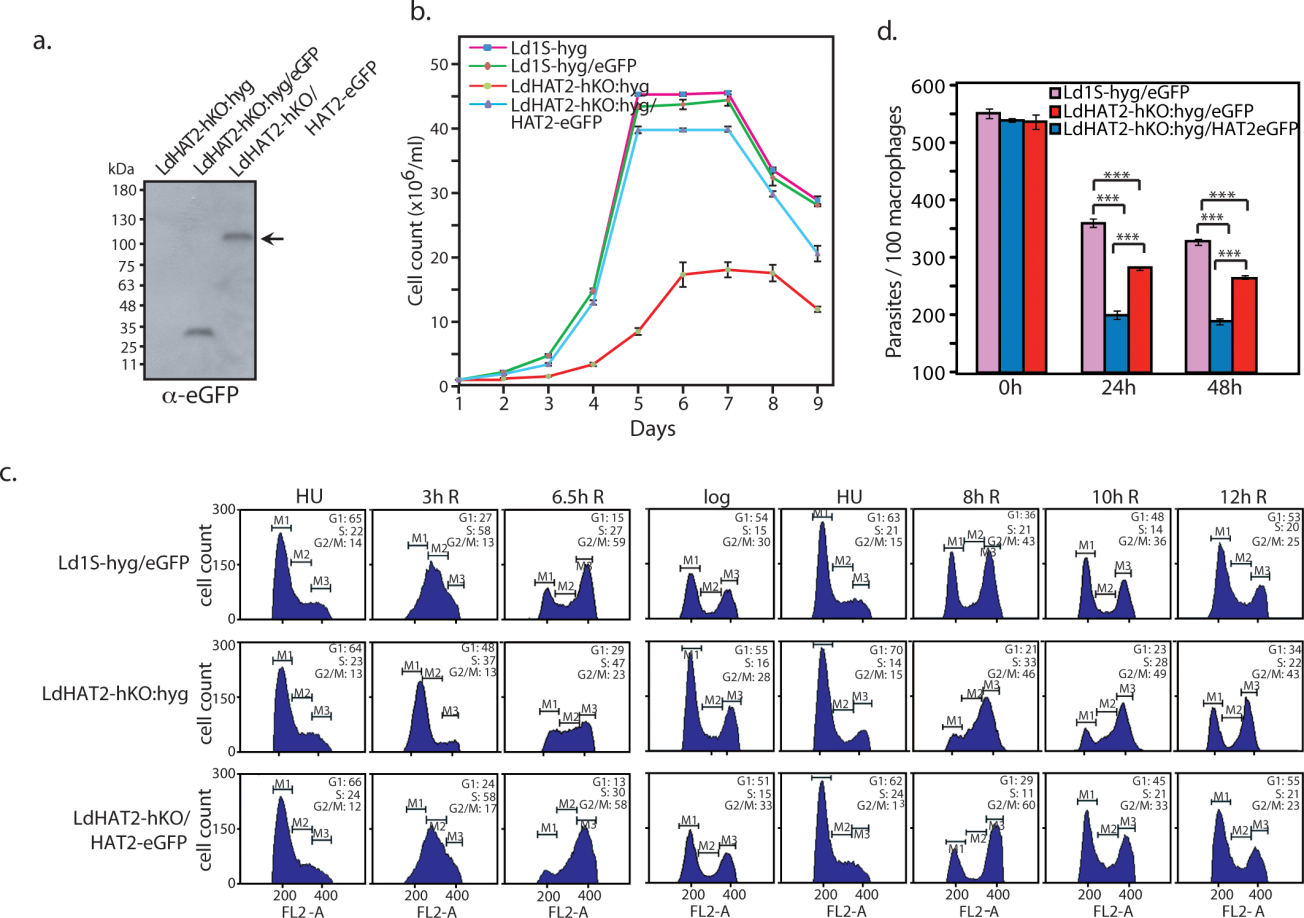
Ectopic expression of HAT2 rescues defects of HAT2-depleted cells. **a.** Western blot analysis of whole cell lysates isolated from LdHAT2-hKO cells and LdHAT2-hKO cells expressing either eGFP or HAT2-eGFP (6x10^7^ cell equivalents per well) using anti-eGFP antibodies (1:2000 dilution). **b.** Growth analysis of rescue line LdHAT2-hKO:hyg/HAT2-eGFP in comparison with LdHAT2-hKO:hyg and control lines Ld1S-hyg and Ld1S-hyg/eGFP. Three experiments were initiated in parallel. Data shown is the average of three experiments, error bars represent standard deviation. **c.** Comparative flow cytometry analysis of HU-synchronized promastigotes of rescue line versus HAT2 heterozygous knockout and control line. Time after release at which sampling was done is indicated above each column of histograms. 30,000 events were analyzed at every time-point, and M1, M2 and M3 gates indicate G1, S and G2/M phases. The percent of cells at different cell cycle stages are indicated in the insets of each histogram. Data set of one of the three experiments performed is shown. **d.** Effect of ectopic expression of HAT2-eGFP on HAT2-hKO parasite propagation/survival within macrophages. Parasites were scored by DAPI-staining of infected cells followed by Z-stack imaging using confocal microscopy. Three separate experiments were initiated in parallel. Bar chart represents average values of three experiments with error bars indicating standard deviation. Two-tailed student’s t-test was applied: ***p < 0.0005.

Possible links between increase in generation time and perturbation of one or more cell cycle stage in LdHAT2-hKO cells were probed by synchronizing cells at the G1/S transition using hydroxyurea and then releasing them into S phase. This enabled the monitoring of cell cycle progression from G1 through S and G2/M, and back into G1 by flow cytometry. Heterozygous knockout cells displayed delay in navigating S and G2/M phases in comparison with control cells, returning to G1 hours later (Fig 2D and 2E). Probing of DNA replication pattern by pulse-labelling cells with EdU at different time-points after release from hydroxyurea block indicated that DNA replication was protracted in heterozygous knockout cells in comparison with wild type (Fig 2F). This is in keeping with findings in *Trypanosoma cruzi* where overexpression of histone H4 that is non-acetylatable at the 10^th^ and 14^th^ residues leads to reduced uptake of EU and EdU, suggesting that H4K10 acetylation is important for replication and transcription [15]. The survival of LdHAT2-hKO:hyg parasites within host cells was examined by infecting macrophages with metacyclic promastigotes [10]. Depletion of HAT2 did not impair their ability to infect macrophages, with the number of parasites per 100 macrophages at the end of the five-hour infection period being the same as in control promastigotes (Fig 2G, 0h time-point). However, the internalized parasites were unable to multiply efficiently within the host cells (Fig 2G, 24h and 48h time-points), underlining the importance of HAT2 for parasite propagation within the mammalian host also. To confirm the defective phenotypes of HAT2-hKO cells were a consequence of HAT2 depletion we expressed LdHAT2 ectopically in the heterozygous knockout cells (Fig 3A shows western blot analysis of lysates isolated from transfectant cells) and analyzed the resulting growth and cell cycle phenotypes. Ectopic HAT2-eGFP expression largely rescued growth and cell cycle defects of HAT2-depleted cells (Fig 3B and 3C), but only partially rescued the heterozygous knockout phenotype when survival within host macrophages was examined (Fig 3D). The reasons for the latter are not understood at the present time.

### Delayed navigation through S phase is due to down regulation of CYC4 in HAT2-depleted cells

**Fig 4:**
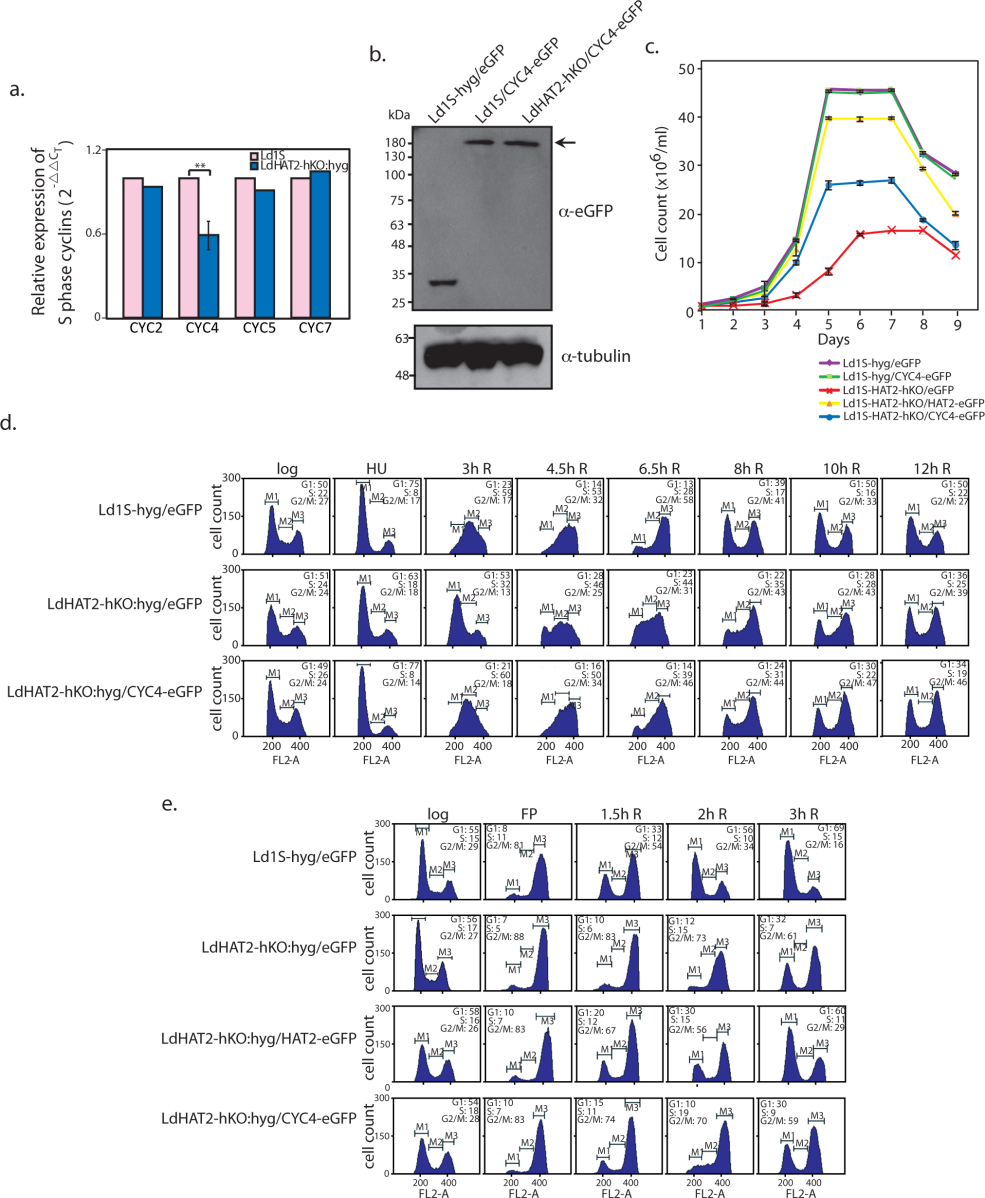
Down regulation of CYC4 causes S phase defects in HAT2-depleted cells. **a.** Analysis of differential expression of S phase cyclins in LdHAT2-hKO:hyg versus Ld1S cells by real time PCR analysis of RNA using 2^-ΔΔC^_T_ method (in which tubulin served as internal control). Two-tailed student’s t-test was applied: **p < 0.005. **b.** Western blot analysis of whole cell lysates isolated from Ld1S-hyg cells expressing either eGFP or CYC4-eGFP and LdHAT2-hKO cells expressing CYC4-eGFP (2x10^8^ cell equivalents per well) using anti-eGFP antibodies (1:2000 dilution). 1/10 of each sample was loaded for tubulin control. **c.** Growth analysis of LdHAT2-hKO:hyg cells expressing CYC4-eGFP ectopically in comparison with LdHAT2-hKO:hyg/HAT2-eGFP rescue line, LdHAT2-hKO:hyg/eGFP heterozygous knockout line, control line Ld1S-hyg/eGFP and Ld1S-hyg cells expressing CYC4-eGFP ectopically. Three separate experiments were initiated in parallel. Values plotted are the average of three experiments, error bars represent standard deviation. **d.** Flow cytometry analysis of HU-synchronized LdHAT2-hKO cells expressing CYC4-eGFP ectopically in comparison with LdHAT2-hKO cells expressing eGFP and control line Ld1S-hyg/eGFP. Time after release at which sampling was done is indicated above each column of histograms. 30,000 events were analyzed at every time-point, and M1, M2 and M3 gates indicate G1, S and G2/M phases. The percent of cells at different cell cycle stages are indicated in the insets of each histogram. Data set of one of the three experiments performed is shown. **e.** Flow cytometry analysis of flavopiridol-synchronized LdHAT2-hKO cells expressing CYC4-eGFP ectopically in comparison with LdHAT2-hKO cells expressing eGFP, LdHAT2-hKO:hyg/HAT2-eGFP rescue line, and control line Ld1S-hyg/eGFP. Data set of one of the three experiments performed is shown.

In considering possible reasons for LdHAT2-hKO:hyg cells moving through S phase and G2/M and then back into G1 later than usual (Fig 2D), we analyzed the expression of the cyclins that govern the transition from G1 through S phase in LdHAT2-hKO cells. RNAi studies have identified four cyclins playing a role in steering cells from G1 through S phase in *T*. *brucei*, with depletion of any of these four cyclins being accompanied by defects in DNA replication [16,17]. Their *Leishmania donovani* orthologs (based on genome sequence annotation; www.tritrypdb.org; [18]) are: CYC2 (LdBPK_320870.1), CYC4 (LdBPK_050710.1), CYC5 (LdBPK_330830.1) and CYC7 (LdBPK_303690.1). On analyzing the relative expression of these four cyclins in logarithmically growing LdHAT2-hKO:hyg cells in comparison with wild type cells, it was observed that CYC4 expression was downregulated to ~0.6 of wild type levels (Fig 4A). To address the possibility of this being the cause of defects in cell cycle progression the CYC4 gene was expressed episomally in HAT2-depleted cells (GenBank Accession No. KY445836 and S1 Methods; Fig 4B shows western blot analysis of lysates from transfectant cells), and the resultant phenotypes analyzed. Episomal expression of CYC4 only partially rescued the growth defects of LdHAT2-hKO cells, and ectopic expression of CYC4 in Ld1S-hyg cells had no impact on growth (Fig 4C). CYC4 (ectopic) expression had a limited impact on cell cycle progression of LdHAT2-hKO cells—by flow cytometry analyses of hydroxyurea-synchronized promastigotes, it was observed that upon release from the HU block LdHAT2-hKO/CYC4-eGFP promastigotes (HAT2-depleted cells expressing CYC4 ectopically) traversed S phase and reached G2/M in timely manner comparable to control cells (Ld1S-hyg/eGFP; Fig 4D). However, they showed prolonged G2/M and delayed re-entry into G1 as did the heterozygous knockout cells. This finding was confirmed by synchronizing cells at G2/M using flavopiridol and then releasing them into G1. The data obtained (Fig 4E) reaffirmed our conclusion that while ectopic expression of CYC4 permitted HAT2-depleted cells to move through S phase smoothly, the cells still displayed delayed navigation through G2/M back into G1. Thus, HAT2 depletion was impacting an additional branch of cell cycle regulation.

### Prolonged G2/M and/or delayed re-entry into G1 in HAT2-depleted cells is due to down regulation of CYC9

**Fig 5:**
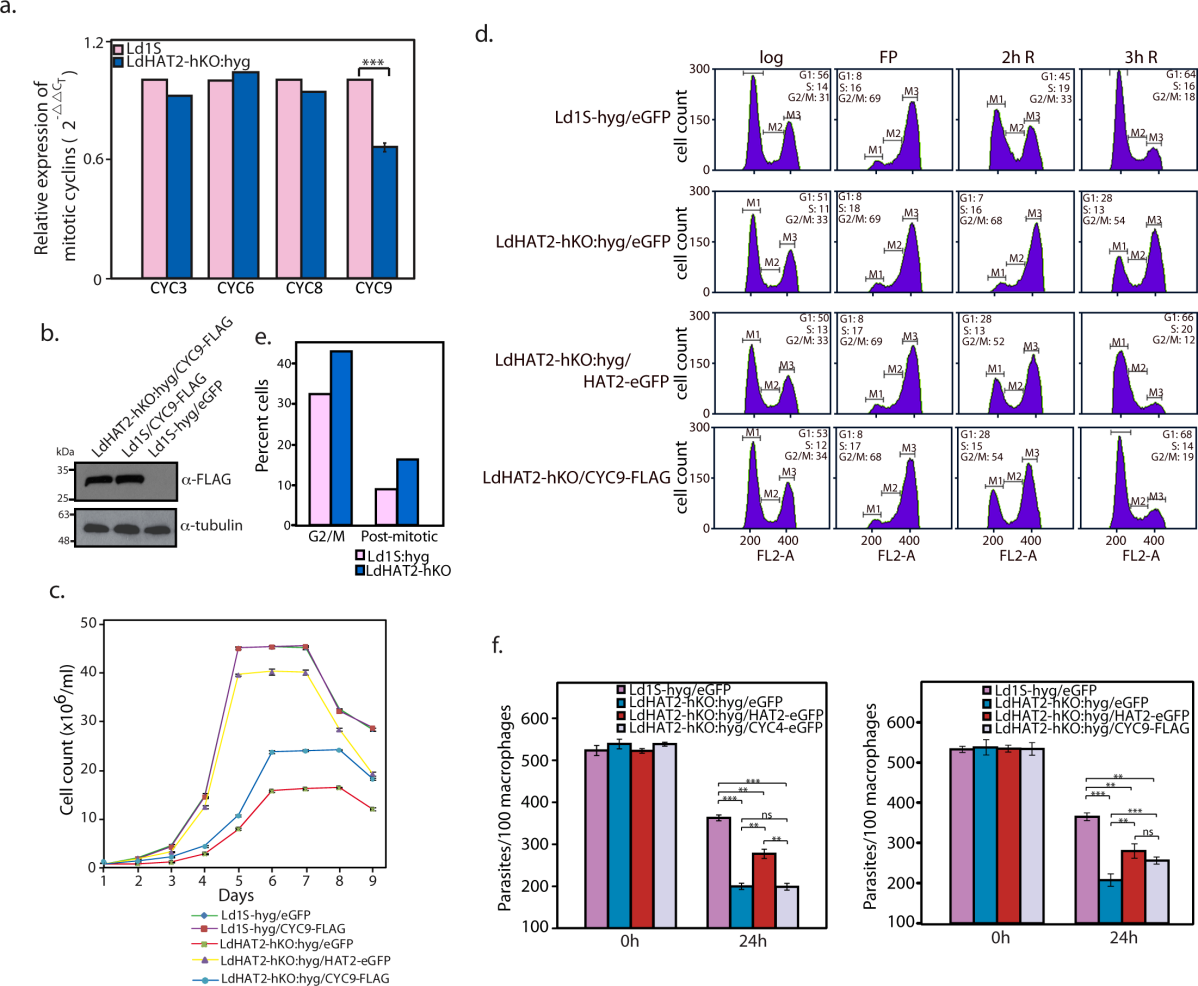
Down regulation of CYC9 causes G2M/post-mitotic defects in HAT2-depleted cells. **a.** Analysis of differential expression of putative mitotic/post-mitotic cyclins in LdHAT2-hKO:hyg versus Ld1S by real time PCR analysis of RNA using 2^-ΔΔC^_T_ method (in which tubulin served as internal control). Two-tailed student’s t-test was applied: ***p < 0.0005. **b.** Western blot analysis of whole cell lysates isolated from Ld1S-hyg cells expressing either eGFP or CYC9-FLAG and LdHAT2-hKO cells expressing CYC9-FLAG (1x10^9^ cell equivalents per well) using anti-FLAG antibodies (1:1000 dilution, Sigma Aldrich). 1/10 of each sample was loaded for tubulin control. **c.** Growth analysis of LdHAT2-hKO:hyg cells expressing CYC9-FLAG ectopically in comparison with LdHAT2-hKO:hyg/HAT2-eGFP rescue line, LdHAT2-hKO:hyg/eGFP heterozygous knockout line, control line Ld1S-hyg/eGFP, and Ld1S-hyg cells expressing CYC9-FLAG ectopically. Three separate experiments were initiated in parallel. Values plotted are the average of three experiments, error bars represent standard deviation. **d.** Flow cytometry analysis of flavopiridol-synchronized LdHAT2-hKO cells expressing CYC9-FLAG ectopically in comparison with LdHAT2-hKO cells expressing eGFP, LdHAT2-hKO:hyg/HAT2-eGFP rescue line, and control line Ld1S-hyg/eGFP. Data set of one of the three experiments performed is shown. **e.** Cells were examined microscopically after DAPI staining to determine the percent of cells in G2/M (2N1K and 1N2K) and the number of post-mitotic cells (2N2K). **f.** Effect of ectopic expression of CYC4-eGFP (left panel) or CYC9-FLAG (right panel) on HAT2-hKO parasite propagation/survival within macrophages. Parasites were scored by DAPI-staining of infected cells followed by Z-stack imaging using confocal microscopy. Three separate experiments were initiated in parallel. Bar chart represents average values of three experiments with error bars indicating standard deviation. Two-tailed student’s t-test was applied: *p < 0.05, **p < 0.005, ***p < 0.0005, ns: not significant.

Mitotic cyclins are characterized by the presence of a nine amino acid motif that plays a role in flagging the cyclin for destruction once mitosis is complete. Three mitotic cyclins have been identified in *T*. *brucei–*CYC3, CYC6 and CYC8. Depletion of either CYC6 or CYC8 (by RNAi) in *T*. *brucei* is coupled to mitotic defects, with the appearance of zoids, and in case of CYC6 knockdown, cells with one nucleus but multiple kinetoplasts as well [19–21]. Additionally, CYC9 has been identified as an essential cyclin of *T*. *brucei*, with RNAi-mediated knockdown of CYC9 resulting in cytokinesis defects in the bloodstream form of the parasite though it has no effect on the procyclic form [22]. The possibility of altered expression of one or more of these four cyclins being responsible for perturbation of G2/M and/or cytokinesis in LdHAT2-hKO:hyg cells was examined by analyzing the expression of the *Leishmania donovani* orthologs of these four cyclins—as annotated in the *Leishmania donovani* strain BPK282A1 genome sequence: LdBPK_300080.1 (CYC3), LdBPK_323520.1 (CYC6), LdBPK_260320.1 (CYC8), and LdBPK_320800.1 (CYC9). Analysis of RNA isolated from logarithmically growing cells revealed that only one of the four was significantly downregulated in HAT2-depleted cells—CYC9 (Fig 5A). We attempted to rescue G2/M and/or cytokinesis defects associated with HAT2 depletion by expressing the Ld1S CYC9 gene (GenBank Accession No. KY445837) in fusion with FLAG tag in these cells (S1 Methods). After confirming expression of CYC9-FLAG in LdHAT2-hKO:hyg/CYC9-FLAG cells (Fig 5B shows western blot analysis of lysates of transfectant cells), when their growth pattern was analyzed it was found that growth defects were partially alleviated (Fig 5C; LdHAT2-hKO:hyg/CYC9-FLAG cells). Flow cytometry analysis of flavopiridol-synchronized cells revealed that G2/M (and/or cytokinesis) defects were alleviated by CYC9-FLAG expression to the same extent as by ectopic expression of HAT2-eGFP (Fig 5D). To determine if CYC9 down regulation in HAT2-depleted cells was accompanied by post-mitotic defects that were delaying re-entry into G1, we analyzed HAT2-depleted cells 8 hours after release from HU-induced block, in comparison to similarly treated Ld1S-hyg cells. Microscopic observation of DAPI-stained cells revealed that almost twice as many cells with two nuclei and two kinetoplasts (2N2K) accumulated in heterozygous knockout cells in comparison to control cells (16% versus 8.8% of each cell type scored; Fig 5E). This suggests that CYC9 down regulation is accompanied by post-mitotic defects linked to delay in cytokinesis, and thus delayed re-entry into G1. When the effect of ectopic expression of CYC4 or CYC9 in LdHAT2-hKO cells on parasite survival/propagation within macrophages was examined, we observed that CYC4 expression did not improve parasite survival within the host cells (Fig 5F; left panel). On the other hand, CYC9 expression alleviated survival defects to the same extent as did ectopic expression of HAT2-eGFP (Fig 5F; right panel). This suggests that CYC9 is relevant to the parasite in both, the promastigote as well as intracellular stage.

### Investigating the possible mechanism by which CYC4 and CYC9 expression is being down regulated in HAT2-depleted cells

**Fig 6:**
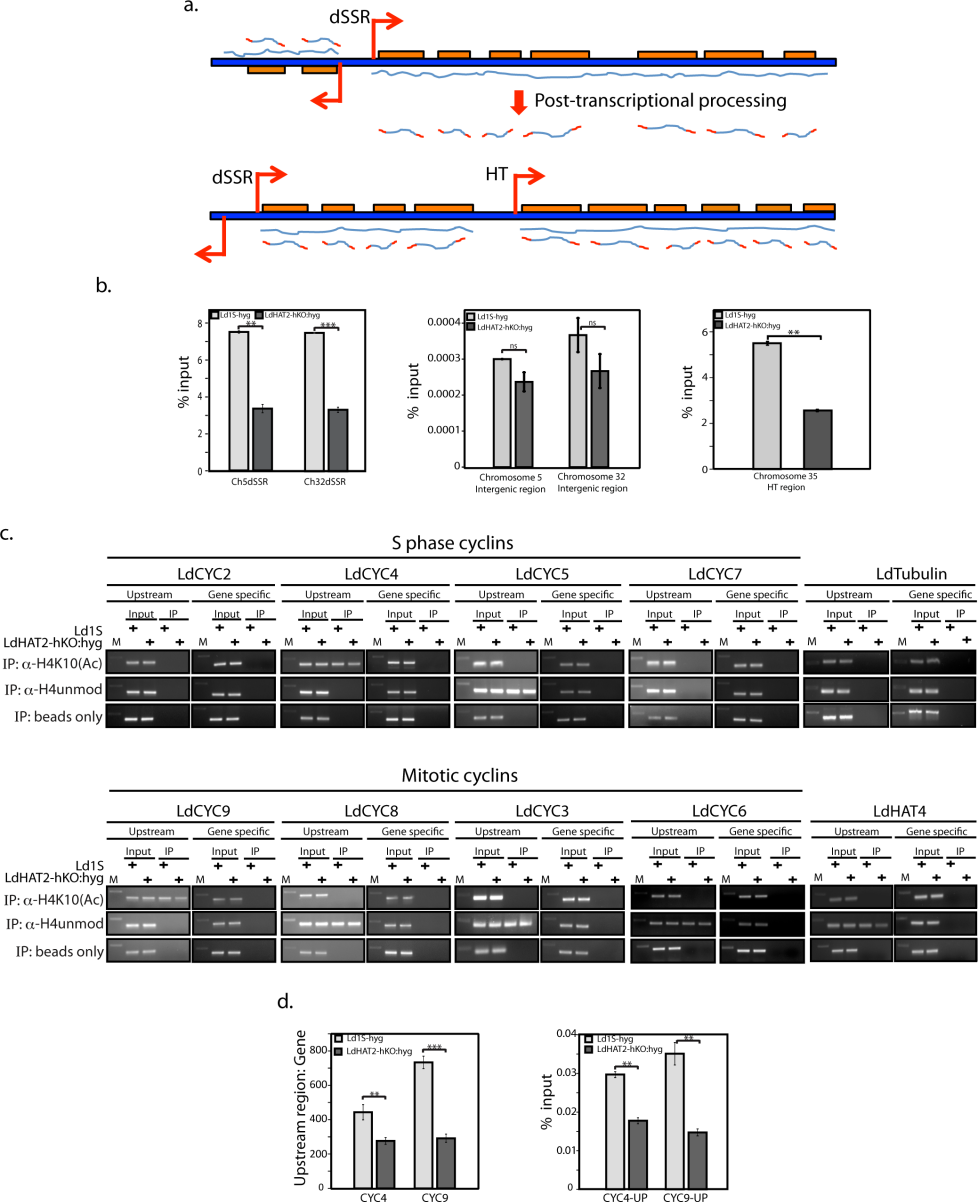
Regions upstream of CYC4 and CYC9 carry H4K10 acetylation. **a.** Schematic representation of mode of transcription in trypanosomatids. dSSR: divergent strand switch region; HT: head-tail. Orange boxes: genes. Red arrowheads indicate direction of transcription. **b, c & d:** ChIP analysis of logarithmically growing asynchronous Ld1S-hyg and LdHAT2-hKO cells. **b.** using anti-H4acetylK10 antibodies, by real time PCR coupled to percent input method. First panel: analysis of regions within chromosome 5 and chromosome 32 dSSRs (Ch5dSSR and Ch32dSSR respectively). Second panel: analysis of non-dSSR intergenic regions within chromosome 5 and chromosome 32. Third panel: analysis of chromosome 35 HT region. **c.** using anti-H4acetylK10 and anti-H4 unmodified antibodies, by PCRs followed by agarose gel electrophoresis. Regions upstream of and within eight cyclin genes, tubulin, and HAT4, were analyzed. Mock-IP: beads only. **d.** using anti-H4acetylK10 antibodies, by real time PCR coupled to percent input method. Regions upstream of and within CYC4 and CYC9 genes were analyzed. Representation of the data along with mock reactions is shown in S6C Fig. In each case three ChIP experiments were carried out and analyzed. In each experiment real time PCR reactions were set up in triplicate. For each experiment the average value for each reaction was determined. Bar chart values presented here represent the mean of the three averages. Error bars indicate standard deviation. Two-tailed student’s t-test was applied: **p < 0.005, ***p < 0.0005.

Towards investigating the mechanism by which HAT2 depletion was down regulating CYC4 and CYC9 expression, we first examined the H4K10 acetylation status of putative promoters in LdHAT2-hKO versus control cells. In trypanosomatid genomes functionally unrelated genes are clustered together unidirectionally, and their transcription occurs polycistronically, followed by posttranscriptional processing of these long pre-mRNA units into mature transcripts prior to translation, by trans-splicing of a 39-nucleotide leader sequence at the 5’ end and subsequent polyadenylation at the 3’ end of individual gene units on the pre-mRNA. The initiation of transcription of the polycistronic transcription units (PTUs) occurs at divergent strand switch regions (dSSRs) on the chromosomes in a majority of cases, with transcription initiating bidirectionally on opposite strands. In a few cases transcription initiates in head-tail (HT) regions, with transcription of a group of genes initiating adjacent to where transcription of the neighbouring group of genes terminates, on the same strand [23–27] (Fig 6A). Transcription initiation site of a PTU may also lie at one end of the chromosome.

H3 acetylation peaks have been detected in dSSRs in *Leishmania major*, and H3K4 methylation and H4K10 acetylation peaks have been detected in dSSRs in *Trypanosoma* species [28–30]. The assessment of the role of H4K10 acetylation in modulating *Leishmania donovani* gene transcriptional events was initiated by probing two dSSRs, one on each of the chromosomes carrying CYC4 and CYC9 genes (chromosomes 5 and 32 respectively; locations indicated in genome map in S4 Fig), in logarithmically growing Ld1S-hyg and LdHAT2-hKO:hyg cells. When chromatin immunoprecipitations were performed with the H4acetylK10 antibody (Materials and Methods), H4K10 acetylation was found to be enriched at both the dSSRs in Ld1S-hyg as well as LdHAT2-hKO cells, with the levels of acetylation being lower in heterozygous knockout cells (Fig 6B, first panel). On analyzing non-dSSR intergenic regions in chromosomes 5 and 32 they were found to be devoid of H4K10 acetylation in both cell types (Fig 6B, second panel). Analysis of the HT region on chromosome 35 that was previously identified as an HT region in *Leishmania major* [31] (location indicated on chromosome 35 genome map in S4 Fig) revealed that here too there was an enrichment of H4K10 acetylation, almost to the same extent as at the dSSRs, with decreased levels of acetylation in HAT2-depleted cells (Fig 6B, third panel). If decreased H4K10 acetylation at dSSRs was indeed the cause of down regulation of CYC4 and CYC9 genes, all the genes lying in the same clusters (PTUs) would be expected to be down regulated. To check this as well as to analyze the global effects of decreased H4K10 acetylation on transcription, microarray analysis of mRNA was carried out (Materials and Methods). To our surprise, we found that genes in the same PTUs were not coordinately up/down regulated in LdHAT2-hKO cells (data submitted to GEO: Accession number GSE76574). Only ~220 genes were downregulated while ~ 440 genes were upregulated, 1.4-fold or more in heterozygous knockout compared to control cells (S4 and S5 Tables). The differentially regulated genes were scattered throughout the genome. In no case was an entire PTU up/down-regulated. In multiple cases there were both, up-regulated and down-regulated genes lying within the same PTU. The coordinately-regulated (either down- or up-regulated) genes were clustered together in only some cases, with ~ 35 dicistronic clusters, ~ 10 tricistronic clusters, and ~ 4 tetracistronic clusters overall from among the ~660 genes that were up/down-regulated (locations of downregulated genes indicated in green in genome map of S4 Fig). It is possible that some of these clusters, particularly the tetracistronics, are coordinately transcribed from head-tail (HT) sites lying internally. Interestingly, none of the five genes that were downregulated in the PTU harbouring CYC9 lay adjacent to each other. On the other hand, among the four genes that were downregulated in the PTU carrying CYC4, one was located immediately downstream of the CYC4 gene—suggesting the possibility of the two genes being coordinately regulated. To address the possibility of the downregulation of the various genes in HAT2-depleted cells being secondary effects of CYC4 and/or CYC9 depletion we co-expressed CYC4-eGFP and CYC9-FLAG ectopically in LdHAT2-hKO cells (as described in S1 Methods; western blot analysis showing co-expression of the two proteins is depicted in S5A Fig) and analyzed the expression pattern of five unrelated genes that were down regulated in HAT2-depleted cells. We found that while HU-synchronized cells co-expressing CYC4-eGFP and CYC9-FLAG ectopically (LdHAT2-hKO/CYC4-eGFP:CYC9-FLAG cells) displayed a similar pattern of cell cycle progression as LdHAT2-hKO:hyg/HAT2-eGFP cells, moving through S phase and G2/M back into G1 in a comparable manner (S5B Fig), these five genes remained downregulated (S5C Fig), indicating that their regulation was independent of CYC4 and CYC9 levels in the cell. Based on these data it is difficult to definitively assert that H4K10 acetylation impacts transcription globally–as these were only heterozygous knockout cells where H4K10 acetylation was not completely abolished, the extent of H4K10 acetylation occurring at the dSSRs and HT sites in these cells (as seen in two dSSRs and the chromosome 35 HT site in LdHAT2-hKO:hyg cells in Fig 6B) was possibly sufficient to permit transcriptional activation from dSSRs and HT sites. The complete absence of H4K10 acetylation might, on the other hand, lead to a more global effect on transcription.

The microarray data confirmed that CYC4 and CYC9 expression levels were down regulated ~1.6-fold and ~1.45-fold (respectively) in HAT2-depleted cells while expression of the other cyclins remained unaffected (S4 and S5 Tables). However, in light of the fact that expression levels of all the genes clustered with CYC4 and CYC9 on the same PTUs were not altered we considered the possibility of H4K10 acetylation in the vicinity of the genes themselves playing a role in the regulation of their expression. ChIPs were performed with H4acetylK10 antibodies as well as antibodies to unmodified H4 using logarithmically growing promastigotes (HAT2-depleted and control cells), and the regions immediately upstream of as well as within the eight cyclin genes, tubulin gene, and HAT4 gene, were analyzed by PCRs followed by gel electrophoresis. H4K10 acetylation was detectable only upstream of CYC4 and CYC9 in both cell types (Fig 6C; “upstream” lanes). H4 with unmodified N-terminus was detected only upstream of CYC3, CYC5, CYC6, CYC8, and HAT4: suggesting the presence of modifications other than H4acetylK10 in the upstream regions of the other genes. These modifications could include H4K4 acetylation, H4K2 acetylation, or H4K14 acetylation, and would preclude the interaction of the anti-H4unmod antibodies (which are directed to the unmodified H4 N-terminus peptide and do not cross-react with the H4 N-terminus acetylated peptides) with H4. No H4K10 acetylation or H4 with unmodified N-terminus was detected within any of the genes (Fig 6C; “gene specific” lanes). The extent of H4K10 acetylation upstream of the CYC4 and CYC9 genes in LdHAT2-hKO versus wild type cells was estimated by ChIP analyses of these two regions using the percent input method. In agreement with the data in Fig 6C, the extent of acetylation at the upstream regions was several hundred-fold higher than within the genes (Fig 6D: first panel). However, while H4K10 acetylation at the upstream regions of both genes was lower in HAT2-depleted cells than in control cells, the extent of this acetylation *per se* was two hundred-fold lower than that seen at the dSSRs and HT region (Fig 6D: second panel compared with Fig 6B: first and third panels), though hundred-fold higher than the non-dSSR intergenic regions (Fig 6D: second panel compared with Fig 6B: second panel).

**Fig 7:**
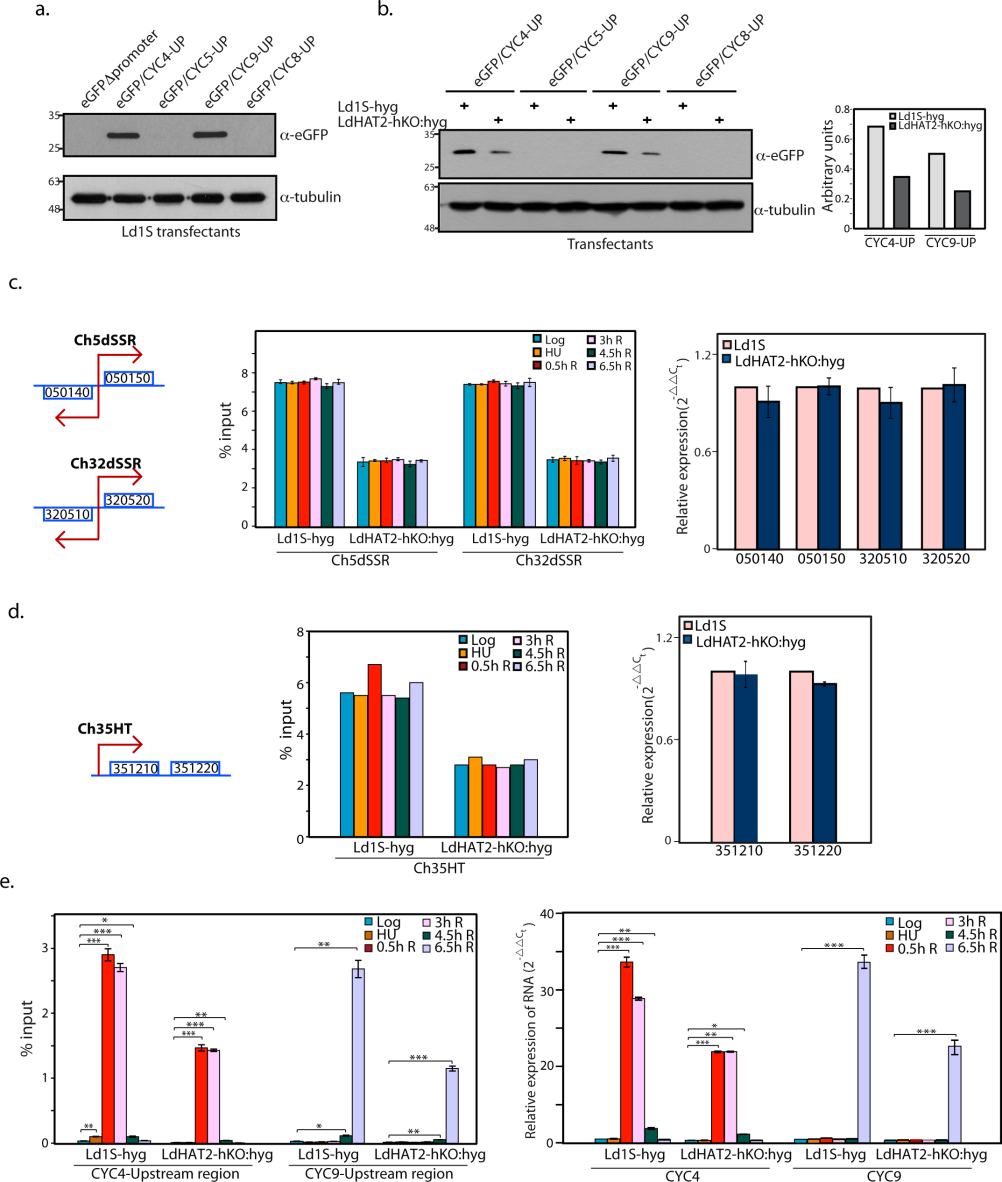
CYC4 and CYC9 promoters are activated in cell cycle-dependent manner. **a.** Western blot analysis of whole cell lysates isolated from Ld1S transfectants (6x10^8^ cell equivalents per well)—using anti-eGFP antibodies (1:2000 dilution). 1/12 of each sample was loaded for tubulin control. **b.** Left panel—Western blot analysis of whole cell lysates isolated from Ld1S-hyg and LdHAT2-hKO transfectants (2x10^8^ cell equivalents per well)—using anti-eGFP antibodies (1:2000 dilution). 1/8 of each sample was loaded for tubulin control. Right panel—ratio of eGFP: tubulin in case of reactions showing eGFP expression, as determined by quantification using ImageJ 1.46r software (Wayne Rasband, NIH, USA). **c, d, e. Left panels:** ChIP analyses of HU-synchronized Ld1S-hyg and LdHAT2-hKO cells using anti-H4acetylK10 antibodies, by real time PCR coupled to percent input method. Analyses were carried out at various time-points, as indicated at upper corner of boxes. ChIP analyses were carried out thrice. In each experiment real time PCR reactions were set up in triplicate, and average value determined. Bar chart values presented here represent the mean of the three averages. Error bars indicate standard deviation. Two-tailed student’s t-test was applied: *p < 0.05, **p < 0.005, ***p < 0.0005. **Right panels:** Analysis of expression of genes in Ld1S-hyg and LdHAT2-hKO:hyg cells by real time PCRs using 2^-ΔΔC^_T_ method (tubulin served as internal control). Three rounds of analyses were carried out, and in each round reactions were set up in duplicate and the average determined. Bar chart values represent the mean of the three averages. Error bars indicate standard deviation. Two-tailed student’s t-test was applied: *p < 0.05, **p < 0.005, ***p < 0.0005. **c.** Left panel: ChIP analysis of regions within chromosome 5 and chromosome 32 dSSRs (Ch5dSSR and Ch32dSSR respectively) Right panel: Gene expression analysis of genes coupled to the dSSRs that were analyzed by ChIP, in logarithmically growing cells. 050140:putative nucleolar RNA helicase II; 050150:hypothetical protein; 320510:hypothetical protein; 320520: Ras-related protein Rab4. **d:** Left panel: ChIP analysis of chromosome 35 HT region. Right panel: Gene expression analysis of genes coupled to the HT that was analyzed by ChIP, in logarithmically growing cells. 351210:pre-mRNA splicing factor ATP-dependent RNA helicase; 351220:arginine-rich protein. **e.** Left panel: ChIP analysis of regions upstream of CYC4 and CYC9 genes. Representation of the data using a log_10_ y-axis is shown in S6D Fig. Right panel: Analysis of expression of CYC4 and CYC9 in HU-synchronized cells, at various time-points as indicated at upper corner of box. Flow cytometry profiles of cells are presented in S6E Fig. Relative expression was analyzed with reference to expression in logarithmically growing asynchronous Ld1S-hyg cells.

Although the degrees of H4K10 acetylation at the regions upstream of CYC4 and CYC9 were much lower than at the dSSRs in a logarithmically growing asynchronous population, the fact remained that no H4K10 acetylation was detectable upstream of the other cyclin genes (Fig 6C). In light of this data along with the fact that genes that were clustered together were generally not coordinately down/up-regulated in LdHAT2-hKO cells (S4 and S5 Tables), we considered the possibility of the regions upstream of CYC4 and CYC9 themselves serving as promoters for the genes. Accordingly, the 1kb regions immediately upstream of the start codons of CYC4, CYC5, CYC8 and CYC9 (S6A Fig) were cloned upstream of the eGFP gene in plasmid pLEXSY_I-egfp-neo3 (as described in S1 Methods), such that they replaced the eGFP promoter region (the deleted region comprised the T7 promoter and utr1 region upstream of the eGFP gene in the pLEXSY plasmid; S6A Fig). The resultant plasmids were transfected into Ld1S promastigotes, and after maintaining selection pressure on polyclonal transfection mixes for two weeks, whole cell lysates were isolated for western blot analysis using anti-eGFP antibodies. We found that eGFP was expressed only from the CYC4 and CYC9 upstream regions (Fig 7A). In comparing this expression in HAT2-depleted cells relative to Ld1S-hyg cells, it was confirmed that eGFP was expressed only from upstream regions of CYC4 and CYC9, with expression being lower in case of HAT2-depleted cells (Fig 7B). Direct fluorescence microscopic analyses also confirmed that expression was downregulated in HAT2-depleted cells, and only detected with CYC4 and CYC9 upstream regions (S6B Fig). Taken together these data suggest that while the CYC4 and CYC9 upstream regions that were analyzed harboured promoter regions, the regions upstream of CYC5 and CYC8 did not. However, the possibility of non-expression of eGFP when the gene is coupled to CYC5 and CYC8 upstream regions being due to post-transcriptional eGFP mRNA processing artifacts cannot be ruled out.

The CYC9 gene on chromosome 32 is located 27 genes downstream of the dSSR, and the nearest down-regulated gene in the CYC9 PTU was ~15 genes downstream of CYC9. Similarly, the CYC4 gene on chromosome 5 is several genes downstream of the putative transcription initiation site of the PTU, and only the gene immediately downstream of it was also downregulated in HAT2-hKO cells. In the light of these facts, the data from the eGFP reporter assays suggested that the CYC4 and CYC9 genes had their own promoters, and these promoters were in addition to the dSSRs from which they were being transcribed as part of PTUs. Thus, we considered the possibility that a second tier of transcriptional activation exists in case of some genes: CYC4 and CYC9 genes among the cyclin genes we examined. Bearing in mind that H4K10 acetylation at the CYC4 and CYC9 promoter regions was ~ 200 fold lower than at the dSSRs on the same chromosomes in logarithmically growing cells (comparing Fig 6B and 6D), we investigated if this possible second level of transcriptional control is cell cycle stage-specific. Accordingly, Ld1S/hyg and LdHAT2-hKO:hyg cells were synchronized with hydroxyurea and cells harvested for ChIP analyses just after the 8 h-long HU-induced block (at G1/S), half an hour after release, 3 hours after release, 4.5 hours after release, and 6.5 hours after release. The two dSSRs on chromosome 5 and 32 (that we had examined in Fig 6) showed equivalent levels of H4K10 acetylation at all times in both cell types, with acetylation levels being overall lower in LdHAT2-hKO cells (Fig 7C, left panel). This was in keeping with previous studies in trypanosomatids reporting H4K10 acetylation peaks at dSSRs and the prevailing understanding that transcription initiation from dSSRs is globally constitutive [24,32,33]. The same pattern of H4K10 acetylation was observed in case of the chromosome 35 HT region as well (Fig 7D, left panel). Contrastingly, H4K10 acetylation levels at the CYC4 and CYC9 promoters varied across the different time-points. At the CYC4 promoter H4K10 acetylation was maximally detected starting at 0.5h after release from block, increasing ~100-fold over levels detected in an asynchronous population (Fig 7E, left panel). These levels of acetylation persisted at 3 hours after release. The acetylation levels dropped subsequently (4.5h R, 6.5h R, Fig 7E, left panel). In case of the CYC9 promoter on the other hand, H4K10 acetylation levels remained low in HU-blocked cells and at 0.5h and 3h after release from block, with slight increase in acetylation at 4.5 hours after release and maximal levels of acetylation (~100-fold greater than that detected in an asynchronous population) being detected 6.5 hours after release (4.5h R, 6.5h R, Fig 7E, left panel). The levels of acetylation in HAT2-depleted cells were about 50% that detected in control cells. These results suggested that the CYC4 and CYC9 promoters, unlike the dSSRs, were activated in a cell cycle -distinctive manner that allowed the upregulation of expression of these two genes around the times at which their products were relevant to the cell. To evaluate this hypothesis we isolated RNA from synchronized Ld1S-hyg and LdHAT2-hKO promastigotes at each of these time-points, and analyzed expression of CYC4 and CYC9 at these stages in comparison with asynchronously growing Ld1S/hyg cells. Flow cytometry profiles revealed that both cell types (Ld1S-hyg and LdHAT2-hKO) had entered S phase in similar pattern by 0.5h after release. However, subsequent to entry into S phase the LdHAT2-hKO cells slowed down, taking much longer to move through S phase (S6E Fig). RNA analyses (Fig 7E, right panel) showed that CYC4 expression in Ld1S/hyg cells was ~50-fold higher 30 min after release from HU-induced block in comparison with expression in asynchronous cells. High levels of expression were maintained 3 hours after release also, following which expression lowered. CYC9 expression, on the other hand, was ~50-fold higher at 6.5 hours after release in comparison with expression in asynchronous cells. Expression of both genes in HAT2-depleted cells was ~50–60% the levels detected in control cells. Considering that HAT2-depleted cells reach G2/M much later than wild-type cells, it is possible that CYC9 expression actually begins to peak earlier than 6.5 hours after release in wild type cells (though after 4.5h post-release). The impact the lower expression of these cyclins has on cell cycle progression in HAT2-depleted cells, emphasizes the importance of stringent modulation of CYC4 and CYC9 expression in *Leishmania*. When the expression of the genes coupled to the two dSSRs and the HT region were analyzed in wild type as well as HAT2-depleted logarithmically growing cells, no difference in expression levels was observed between the two cell types (Fig 7C, right panel and Fig 7D, right panel respectively), affirming that decreased acetylation at dSSRs and HT region in HAT2-depleted cells did not impact expression of the neighbouring genes. Taken together, these findings supported the inference that the expression of CYC4 and CYC9 genes are tightly regulated by H4K10 acetylation at their individual promoters, with transcriptional activation of the two genes from their promoters being linked to those phases of the cell cycle where they are functionally pertinent.

**Fig 8:**
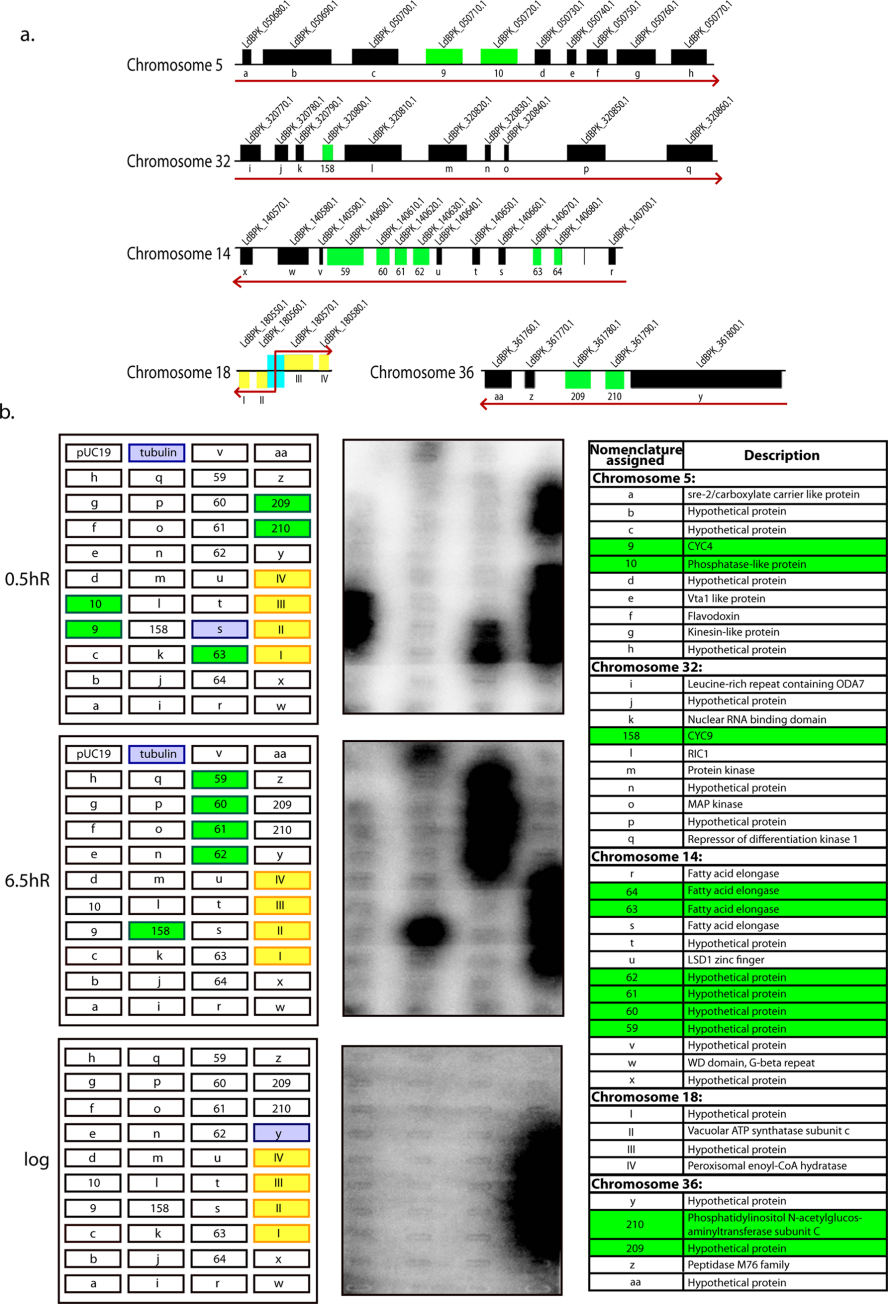
Nuclear run-on assays performed with Ld1S promastigotes. **a.** Schematic representation of the genomic locations of genes being analyzed in run-on transcription assays, adapted from *Leishmania donovani* genomic map available in GeneDB (www.genedb.org; [45]). Genes marked as green boxes are downregulated in HAT2-depleted cells. Genes marked as yellow boxes indicate genes immediately adjacent to a dSSR. Red arrows indicate direction of transcription of PTUs. Downregulated genes carry a designated arabic numeral that corresponds to their serial number in S4 Table, and all other genes carry a designated alphabet. **b. Left panels:** schematic representations of slot blots indicating the blot position of each gene that was analyzed. At each time-point, slots corresponding to transcriptionally activated genes which matched with genes that were downregulated in HAT2-depleted cells are marked green while slots corresponding to transcriptionally activated genes that are linked to a dSSR are marked yellow. Other activated genes are marked mauve. Positive control: tubulin. Negative control: pUC19 plasmid with no insert. **Centre panels:** phosphorimaging of blots after hybridization with radiolabeled nascent RNA isolated from nuclei. Shorter and longer exposures are presented in S7 Fig. The table lists the description of all genes analyzed.

However, these experiments measured steady state RNA levels and therefore there was a possibility that the differential expressions of CYC4 and CYC9 that we were detecting at different cell cycle stages were the outcome of RNA processing and RNA stability effects. To confirm that transcription of these genes was being activated at specific cell cycle stages we carried out nuclear run-on assays using HU-synchronized promastigotes, as detailed in Materials and Methods. Genes at five different regions of the *Leishmania* genome were analyzed (Fig 8A). In analyzing CYC4 and CYC9 transcriptional activation we also examined the three genes immediately upstream and six genes immediately downstream of them, on chromosomes 5 and 32 respectively (Fig 8A, S4 Fig). A section of a chromosome 14 PTU harbouring a dicistronic as well as tetracistronic cluster that were both downregulated in HAT2-depleted cells, and a section of a chromosome 36 PTU harbouring a dicistronic cluster that was downregulated in HAT2-depleted cells (Fig 8A, S4 Table, S4 Fig) were also examined. Additionally, four genes coupled to a chromosome 18 dSSR were analyzed (Fig 8A, S4 Fig). Nuclear run-ons were performed with HU-synchronized cells 0.5 h and 6.5 h after release, as well as with logarithmically growing cells, and the data obtained are presented in Fig 8B.

As evident from the results presented in Fig 8B and S7 Fig, the CYC4 gene as well as the gene immediately downstream of it were both transcriptionally activated 0.5h after release from HU-block, but not 6.5h after release (Fig 8B, S7 Fig). No other genes in the cluster were activated to the same extent at either of the two time-points. In the chromosome 32 cluster that was analyzed CYC9 was transcriptionally activated only at 6.5h after release, and none of the other genes in the cluster were activated to the same extent at either time-point. The genes on chromosome 14 PTU displayed a dichotomous behavior—the tetracistronic cluster that is down regulated upon HAT2 depletion was transcriptionally activated 6.5h after release but not 0.5h after release, while of the two genes in the dicistronic cluster that is downregulated upon HAT2 depletion, only the second was transcriptionally activated, at 0.5h after release. Interestingly, the gene downstream of it was also transcribed actively, though to a lesser extent. The dicistronic cluster on chromosome 36 that is downregulated upon HAT2 depletion was transcriptionally activated only at 0.5h after release. Though CYC4 and its downstream partner both continued to be transcribed even 3 hours after release, the two dicistronic clusters on chromosomes 14 and 36 were no longer transcribed (S7 Fig). Other than the CYC4 dicistronic cluster and the genes coupled to the chromosome 18 dSSR none of the genes were activated at 3 hours after release (S7 Fig).

The remaining genes in all the PTUs analyzed were being transcribed at both time-points (and at 3 hours after release), but to a much lesser extent, as is evident from the longer exposures of the blots (S7 Fig). In looking at logarithmically growing cells none of these genes appeared to be upregulated, probably because in an asynchronous population most cells would be in G1. The genes coupled to the chromosome 18 dSSR were transcriptionally active in all cases, including in a logarithmically growing population (Fig 8B, S7 Fig). Interestingly, these genes were highly transcribed at all times, in contrast to the other analyzed genes (lying within chromosome 5, chromosome 32, chromosome 14, and chromosome 36) that were not activated in stage-specific manner but which were nevertheless transcribing at all times to a lesser extent (S7 Fig). This suggests that transcription may not be activated equally at all dSSRs. In fact, considering the fact that no chromosome 18 genes were downregulated in HAT2-depleted cells, suggesting the absence of any cell cycle stage-specific transcriptional activation of genes on this chromosome, it is possible that PTUs which are transcribed entirely and solely from dSSRs may be more actively transcribed. However, a separate detailed investigation would be necessary to determine if this is indeed so.

These data allowed us to definitively conclude that transcription of CYC4 and CYC9 was being activated at specific times during cell cycle progression. Moreover, transcription of the activated CYC9 gene appears to be terminating after the gene itself, in keeping with the microarray data which showed only CYC9 expression to be downregulated in LdHAT2-hKO cells (Fig 8B, 6.5hR). Likewise, transcription of the activated CYC4 and its adjacent gene appears to terminate after the adjacent gene, again in agreement with the coordinated downregulation seen in microarray data of LdHAT2-hKO cells. It is difficult to conclude if the coordinately activated genes that are clustered together are transcribed from the promoter region upstream of the first gene in the cluster, or each have independently activated promoter regions. Taken together these data demonstrate that transcriptional processes in trypanosomatids are much more complex than was believed to be thus far.

## Discussion

MYST-family HATs are ubiquitously found across eukaryotes, and regulate multiple DNA-related processes. The present study was undertaken with the aim of determining the functional role of *Leishmania donovani* HAT2. The constitutively nuclear LdHAT2 targeted H4K10 for acetylation both *in vitro* and *in vivo*. HAT2-depleted promastigotes displayed slower growth and longer generation time, and exhibited cell cycle and DNA replication defects, also being unable to propagate efficiently within macrophages. Knockdown of HAT2 expression by RNAi in *T*. *brucei* too results in severe growth defects and accumulation of cells pre-cytokinesis but the mechanism by which this is occurring has not been demonstrated [11]. The results of our study indicate that CYC4 and CYC9 downregulation were responsible for cell cycle defects in HAT2-depleted promastigotes.

The unique genome arrangement of kinetoplastids is linked to the coordinated transcription of functionally unrelated genes. In *Leishmania major* nuclear run-on analyses have demonstrated that transcription of the PTUs on chromosome 1 and chromosome 3 initiate bidirectionally on opposite strands in the divergent strand switch regions (dSSRs) [25,26,34], and subsequent results from a genome-wide study in asynchronously growing *Trypanosoma brucei* by Kolev et al [24] showed this to be the primary mode of transcription initiation in trypanosomatids. A few head-tail (HT) transcriptional start sites have also been identified, with the initiation of transcription of a cluster of genes occurring adjacent to the termination of transcription of the neighbouring cluster on the same strand [24,27]. Transcription from dSSRs is viewed as occurring constitutively and uniformly across the PTUs, and steady state levels of mRNA are believed to be the outcome of post-transcriptional processes that govern mRNA stability: for example, heterogeneity in pre-mRNA processing in terms of the sites at which the 5’ leader sequence is spliced and sites at which polyadenylation occurs [24,27,32,33]. The transcription start sites (TSSs) in dSSRs are enriched in H4K10 acetylation and H3K4 trimethylation in *T*. *brucei* and *T*. *cruzi*, and in acetylated H3 in *L*. *major* [27–30,35]. In accordance with these reports, we found dSSRs on the two chromosomes carrying the CYC4 and CYC9 genes to be enriched in H4K10 acetylation in *Leishmania donovani* cells, with levels of this acetylation being lower in HAT2-depleted cells as expected. However, contrary to our expectations, the genes lying in the CYC4 and CYC9 clusters were not all coordinately downregulated in HAT2-depleted cells. Additional peaks of H4K10 acetylation and H3K4 methylation have also been detected at a few non-SSR sites in *T*. *brucei* (asynchronous cultures), and some of these were later shown to be at head-tail (HT) transcriptional start sites [24,27,35]. In *Leishmania major* HT sites have been identified by Lombrana *et al* based on the H3 acetylation peaks and J base peaks identified by Thomas *et al* and van Luenan *et al* respectively [29,31,36] and although 51 sites have been identified, the regions immediately upstream of CYC4 and CYC9 are not among them, nor have any HT sites been identified around the one tetracistronic and two dicistronic clusters that we analyzed in the run-ons.

When we analyzed asynchronous cells, although we detected H4K10 acetylation immediately upstream of CYC4 and CYC9 genes, the extent of acetylation was two hundred-fold lower than at dSSRs, making it difficult to envisage a role in gene regulation although eGFP-based reporter assays suggested that these regions were active promoters. In the light of this data we examined the possibility of the existence of a second tier of activation of gene expression that may be cell cycle stage-specific. The results presented in this study revealed that unlike the dSSRs, and HT region on chromosome 35, which showed equivalent H4K10 acetylation levels at all times, CYC4 and CYC9 promoters displayed stage-specific enrichment of H4K10 acetylation, and this was coupled to upregulated CYC4 and CYC9 mRNA levels due to transcriptional activation. Though H4K10 acetylation was enhanced at the CYC4 and CYC9 promoters at specific times, the maximal levels of acetylation we detected (in wild type control cells) were still only about half the acetylation levels at the dSSRs in these cells, leading us to conclude that the CYC4 and CYC9 promoters are much more sensitive to H4K10 acetylation levels than dSSRs are. In the absence of specific antibodies we have been unable to check the status of H3K4 methylation at the two gene promoters. Promoter elements have remained largely undefined in trypanosomatid species in spite of continuing efforts, with no conserved consensus sequences being identified across dSSRs or HT sites thus far. A recent report proposes that GT-rich promoters help activate transcription in trypanosomatids in a context-dependent manner by modulating the local chromatin environment [37]. We examined the CYC4 and CYC9 upstream regions and compared them with the dSSRs of chromosome 5, chromosome 18 and chromosome 32 as well as the HT site on chromosome 35. While no common sequence motif was identifiable, it was observed that all analyzed sequences harboured homopolymeric tracts (S8 Fig). Previous analysis of the chromosome 1 dSSR of *Leishmania* revealed the presence of a poly(C) tract that was conserved across fifteen *Leishmania* species [38]. It is believed that homopolymeric tracts have a negative impact on nucleosome assembly due to their relative rigidity [39], thus promoting transcription.

**Fig 9:**
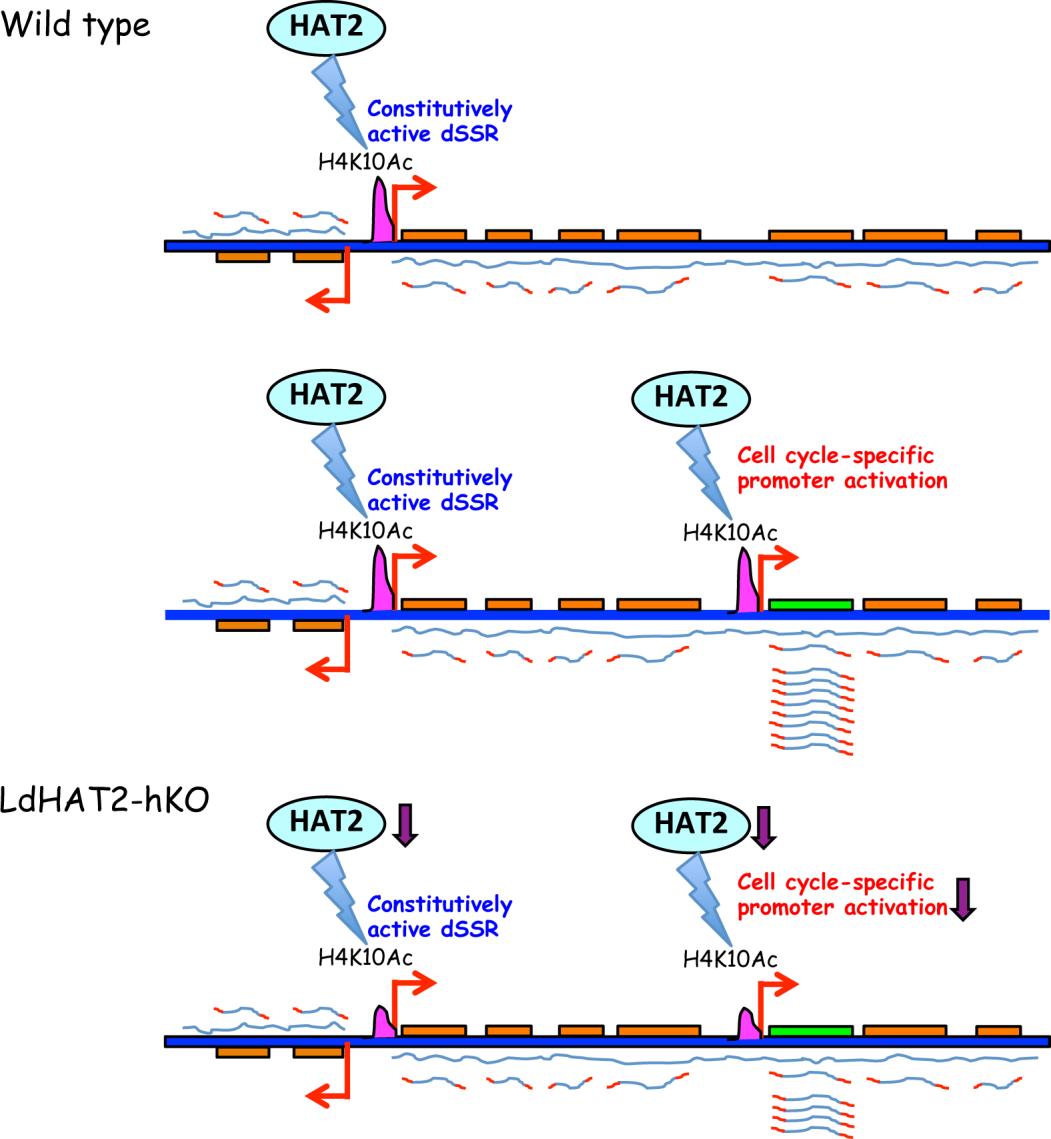
Schematic representation of possible mechanism of gene regulation by *Leishmania donovani* HAT2. Upper panel: HAT2-mediated H4K10 acetylation of dSSRs is constitutively maintained, helps activate global transcription of polycistronic gene clusters that are then processed to individual gene transcripts prior to translation. Middle panel: In addition to dSSR activation, HAT2-mediated H4K10 acetylation activates certain gene-specific promoters in a cell cycle-dependent manner, resulting in dramatic upregulation of the gene’s expression. Lower panel: In HAT2-depleted cells H4K10 acetylation decreases by ~ 50% at both, dSSRs and at cell cycle-distinctive promoters. However, transcription initiation from dSSRs remains largely unaffected, while initiation at cell cycle-distinctive promoters is proportionately lowered, reducing gene expression.

The findings of the present study reinforce the fact that transcriptional regulation in trypanosomatids is a complex process. It appears that there are two levels of control (Fig 9). The dSSRs are the primary sites of transcription initiation, and their activation is believed to be linked to H3 acetylation, H4K10 acetylation and H3K4 methylation as these modifications are enriched at the transcription start sites. From our data it is difficult to attribute a role to H4K10 acetylation in transcriptional activation from dSSRs in *Leishmania donovani* as an ~ 50% decrease in H4K10 acetylation in HAT2-depleted cells at the dSSRs (and HT region) that we examined, did not have an impact on gene expression (Fig 7C and 7D). However, the equivalently high levels of H4K10 acetylation that we detect at dSSRs throughout the cell cycle are in accordance with the prevailing thought that the dSSRs are constitutively active. A small subset of genes are controlled at a second level by gene-specific promoters. This second level of regulation appears to be cell cycle stage-dependent for some genes at least, with promoter activation occurring specifically at or near the time when the proteins encoded by those specific genes are functionally critical to the cell. While decreased levels of H4K10 acetylation at the dSSRs (an ~ 50% decrease) appear to be sufficient to support global gene expression at the same levels as wild type cells, the second tier of regulation at gene-specific promoters is perturbed by HAT2-depletion. This level of control that is more sensitive to H4K10 acetylation levels results in downregulation of expression and consequently the defective phenotypes associated with HAT2-depletion. The results of the nuclear run-on assays indicate that the genes in these PTUs which are not transcriptionally activated at specific cell cycle stages are nevertheless being transcribed at lower levels. As stage-specific transcription is terminating directly downstream of the activated genes it appears that the other genes in the cluster must be transcribing from the dSSRs as part of the PTUs, perhaps constitutively. It is possible that hitherto unidentified accessory proteins are involved in RNA polymerase II-mediated transcription initiation (and elongation) in trypanosomatids. The factors regulating initiation events at internal promoters may not be involved in transcription initiation events at dSSRs, and the transcription complexes transcribing PTUs from dSSRs may differ in composition from the complexes transcribing genes from internal promoters. Previous studies have identified base J enrichment peaks at the sites of transcription termination of PTUs. However, no base J peaks were identified downstream of any of the genes activated from internal promoters that we have analyzed as part of this study [36]. The accessory protein factors that are part of the complexes transcribing from internal promoters may mediate transcription termination immediately downstream of the genes. Much more detailed investigations are necessary to unravel the mechanism(s) by which internal transcriptional activation and termination events are occurring. Future efforts would be directed towards understanding how cell cycle-dependent transcriptional activation within long PTUs results in the upregulation of only one to four genes immediately adjacent to the internal promoter, but not genes beyond them.

A large number of genes are upregulated in HAT2-depleted cells. These increased levels of mRNA may be due to perturbations in posttranscriptional processing in response to the slower growth rate of HAT2-depleted cells. Differential regulation of specific mRNAs at specific cell cycle stages has been seen in only a handful of cases in trypanosomatids so far, and has been put down to being the consequence of post-transcriptional events governing mRNA stability. This report presents the first data suggesting that there are gene-specific promoters lying within the PTUs that are activated in a cell cycle-distinctive manner. Our results underscore the complexities of transcriptional processes in trypanosomatids and add a new dimension to our knowledge of the mechanisms regulating gene expression in these unicellular eukaryotes.

## Materials and methods

### Leishmania cultures

*Leishmania donovani* 1S cells were maintained at 26°C in M199 medium (Lonza) supplemented with 10% fetal bovine serum (Invitrogen), adenine, glutamine and hemin (all from Sigma Aldrich, USA), as described earlier [40]. Details of isolation and fractionation of cell extracts, growth and survival analyses, determination of generation time, synchronization regimes, flow cytometry regimes are given in S1 Methods.

### Histone acetyltranferase (HAT) assay

Histone acetyltransferase assays were performed with HAT2-FLAG proteins (wild type and E332A mutant) pulled down from whole cell lysates isolated from transfectant clonal lines, using the HAT Assay Kit (Active Motif, USA) as detailed earlier [9]. The assay is based on the fluorescence-based detection of the free thiol groups produced on CoA when the acetyl group from acetyl-CoA is transferred to the peptide/protein substrate. In a typical experiment, the FLAG-tagged proteins were pulled down from lysates isolated from ~2x10^10^ promastigotes, using FLAG M2 agarose beads (Sigma Aldrich). The bead-bound protein fraction was then equally divided into five parts, and each part (equivalent to pull down from 4x10^9^ promastigotes) used in a single histone acetyltranferase reaction. In every experiment one part was used to determine autoacetylation levels (reaction performed in absence of any peptide substrate; a measure of the extent of HAT2-mediated self-acetylation) and the other parts were used to determine the sum of autoacetylation and peptide acetylation. The sequences of the peptides (synthesized by Peptron Inc, South Korea or Abgent, USA) used as substrates in these reactions (depicted in the boxes above the bar charts in Fig 1C–1E) corresponded to the sequences of the N-terminal tails of the *Leishmania* core histones H2A, H2B, H3 and H4 as well as the C-terminal tail of H2A.

Reactions were performed as per the manufacturer’s instructions and the free thiol groups produced at the end of the reaction were detected by using the developer (provided in the kit), resulting in the production of fluorescence. The extent of fluorescence obtained (excitation: 360–390 nm, emission: 450–470 nm) was used as a measure of the extent of acetylation that had occurred. Values presented in the bar charts are arbitrary fluorescence units, and have been plotted after subtracting background values (values obtained in reactions carried out in the absence of HAT2 protein and peptide substrate). The data presented in the bar charts are average values of three independent experiments. Error bars indicate standard deviation. Student t-test (two-tailed) was applied to analyze the results, and *P* values are presented in figure legends.

### EdU labeling analysis

EdU labeling analysis was done as detailed earlier [10]. Briefly, Ld1S-hyg and LdHAT2-hKO:hyg promastigotes were synchronized using hydroxyurea, released into drug-free medium, and aliquots of cells pulsed with 5-ethynyl-2-deoxyuridine (EdU) at different time-points after release. EdU-pulsed cells were analyzed microscopically using the Click-iT EdU Imaging Kit (Invitrogen) followed by confocal imaging and analysis as above.

### RNA isolation and analysis by real time PCR

Isolation of RNA, synthesis of cDNA, and analyses of gene expression by real time PCR were carried out using tubulin as internal control as detailed earlier [10], by the 2^-ΔΔC^_T_ method [41]. Primers used for expression analyses are listed in S2 Table. All primers were designed using the *Leishmania donovani* BPK282A1 published sequence.

### DNA microarray analysis

RNA was isolated from Ld1S and LdHAT2-hKO logarithmically growing promastigotes using the PureLink RNA mini kit (Invitrogen), and analyzed using Bioanalyzer to confirm purity and integrity prior to microarray analysis. Microarray analysis of mRNA was performed as detailed earlier [10] using Gene Expression *Leishmania* 8x15K, AMADID:035638 (Genotypic Technology), and data analysis was done using GeneSpring GX Version 12.1. The analysis was carried out using biological replicates (RNA isolated from two separate experiments).

### Macrophage infection experiment

Macrophages were infected with metacyclics as detailed previously [10]. For each experiment, three biological replicates were set up in parallel. Data presented here represents the mean values of the three experiments with error bars depicting standard deviation. Student t-test (two-tailed) was applied to analyze the results, and *P* values are presented in figure legends.

### ChIP analysis

Chromatin immunoprecipitation procedure was adapted from the protocol of Lowell and Cross [42]. *Leishmania* promastigotes (1x10^9^ cells) were resuspended in fresh M199 complete medium (50 ml), and fixed with 1% formaldehyde for 30 min at room temperature. The reaction was stopped by incubation with 125 mM glycine at room temperature for 5 min. Cells were collected by centrifugation, washed twice with 1X PBS, harvested, and resuspended in lysis buffer (50 mM Tris.Cl (pH 8.0), 10 mM EDTA, 1% SDS) containing protease inhibitors and sodium butyrate (50 mM), followed by incubation on ice for 15 min. The cell suspension mix was sonicated on ice (30 sec on/60 sec off), cell debris removed by high speed centrifugation, and supernatant stored in 200 μl aliquots at -20°C. DNA fragment size was confirmed to be between 300–600 bp by reversing cross-links at 65°C overnight, followed by purification of the DNA (using Qiaquick PCR purification kit) and analysis using agarose gel electrophoresis.

For chromatin immunoprecipitations, the input lysates (200–300 μl) were diluted with immunoprecipitation buffer (16.7 mM Tris.Cl (pH 8), 1.2 mM EDTA, 150 mM NaCl, 1.1% Triton-X, 0.01% SDS, 50 mM sodium butyrate, and protease inhibitors) and pre-cleared by incubation with 30 μl protein A-Sepharose beads (Invitrogen) for 90 min at 4°C with rotation. The supernatant was collected following low speed centrifugation, 5 μl antibodies (anti-H4acetylK10 or anti-H4 unmodified) added to it, and incubated overnight at 4°C with rotation. Further addition of 25 μl protein A-sepharose beads was followed by incubation for 90 min at 4°C with rotation. After removal of unbound fraction by low speed centrifugation, the beads were successively washed in four different buffers: Wash Buffer A (20 mM Tris.Cl (pH 8.0), 2 mM EDTA, 150 mM NaCl, 0.1% SDS, 1% Triton-X, protease inhibitors and sodium butyrate), Wash Buffer B (same as Wash Buffer A except 500 mM NaCl), Wash Buffer C (10 mM Tris.Cl (pH 8.0), 1 mM EDTA, 250 mM LiCl, 1% NP-40, 1% sodium deoxycholate, protease inhibitors and sodium butyrate) and Wash Buffer D (TE buffer containing protease inhibitors and sodium butyrate). Each wash included incubating the beads in the wash buffer for 15 min at 4°C with rotation and collecting the wash following low speed centrifugation. The bound fraction was eluted in 0.1 M sodium bicarbonate, 1% SDS (250 μl) by rotation for 10 min at room temperature. Cross-links were reversed after addition of sodium chloride (200 mM) at 65°C overnight. Following RNase treatment for 30 min the DNA in the fraction was purified using the Qiaquick PCR purification kit, and eluted in 10 mM Tris.Cl (pH 8.5). 1/50^th^ of the eluted fraction was used per PCR. ChIP analyses were carried out using real time PCR coupled to the percent input method as described [43], using the appropriate primer pairs designed against the regions under investigation (S3 Table). All ChIP analyses experiments were done three times, and in each experiment samples were analyzed in triplicates. Values presented are the average of three experiments, with error bars depicting standard deviation. No antibody was added in mock reactions which were otherwise carried out exactly as actual reactions.

### Nuclear run-on analysis

Nuclear run-on assays were carried out as described earlier [25,44], with modifications. For each run-on reaction nuclei were isolated from 1x10^9^ promastigotes (either logarithmically growing or synchronized). Harvested cells were washed once with 1X PBS, and resuspended in 2.5 ml ice-cold hypotonic buffer (0.25M sucrose, 5 mM HEPES pH 7.5, 1 mM spermidine, 0.1 mM PMSF, 1 mM EDTA, 1 mM EGTA, 1 mM DTT). Cells were lysed by adding Nonidet P-40 and Triton X-100 (final concentration 0.5% each) and vortexing vigorously for 35 seconds. This was followed by the immediate addition of 5 ml of ice-cold wash buffer (40 mM Tris.Cl pH 7.5, 0.64 M sucrose, 1 mM spermidine, 0.1 mM PMSF, 1 mM EDTA, 1 mM EGTA, 1 mM DTT and 60 mM KCl), quick vortexing to mix, and centrifugation at 3000*g* for 10 minutes at 4°C. After decanting the supernatant the wash was repeated once and this time the nuclei were collected by centrifugation at 1000*g* for 10 minutes at 4°C. The supernatant was removed carefully and 0.1 ml transcription buffer reaction mix (100 mM HEPES pH 7.5, 2 mM MgCl_2_, 4 mM MnCl_2_, 0.15 mM spermine, 0.5 mM spermidine, 50 mM NaCl, 50 mM KCl, 25% glycerol, 2 mM ATP, 2 mM GTP, 2 mM CTP, 100 μCi UTP (α-^32^P UTP, 3000 Ci/mmole, 10 mCi/ml from BRIT, India), 2 mM DTT, 40 U RNasin) added to the pelleted nuclei before tapping to suspend evenly. Reaction was incubated at 26°C for 8 minutes, followed by the addition of 2 mM UTP and further incubation at 26°C for 2 minutes. 25 U of DNase I were added and reaction moved to 37°C for 5 minutes, before the addition of 0.1 ml Stop buffer (10 mM Tris.Cl pH 7.5, 10 mM EDTA, 1% SDS, 200 μg/ml proteinase K solution) and further incubation at 37°C for 5 minutes. Reactions were subjected to phenol: chloroform extraction and the labeled nascent RNA collected by precipitation using ethanol.

Slot blots were prepared, on which the linearized and denatured DNA probes were immobilized (5 μg of each probe clone). The probes comprised of ~1 kb fragments from the 5’ ends of the genes being analyzed, that had been amplified off genomic DNA and cloned into plasmid vector (sequences of primers used to amplify the probe fragments available on request). In case of genes smaller than 1 kb entire genes were used as probes. The blots were pre-hybridized for 6 hours at 42°C in 50% formamide, 4X Denhardt’s solution, 5X SSC and 0.2% SDS, to which 100 μg/ml salmon sperm DNA was added. The labeled nascent RNA was added (2.5x10^6^ cpm/5ml) and hybridization allowed for 72 hours at 42°C. Blots were washed with 2XSSC/0.1% SDS at room temperature for 20 min and again at 42°C for 20 min before exposing the blots for phosphorimaging for 3–10 days.

### Data availability

The authors confirm that all data underlying the findings are fully available without restriction. All DNA microarray files are available from the Gene Expression Omnibus database (accession number GSE76574).

## Supporting information

S1 Methods S1 Methods carries supplementary methods describing *Leishmania* cultures, cloning of HAT2 gene and its expression in *Leishmania* for HAT assays, cloning of CYC4 and CYC9 genes and their expression in *Leishmania* cells, cloning of upstream regions of cyclin genes, raising modification-specific antibodies and analysis of their specificity by peptide competition assays, tagging of HAT2 genomic allele with eGFP, creation of HAT2 knockout and rescue lines, and immunofluorescence analysis. (DOCX)

S1 Table List of primers used for clonings. (DOCX)

S2 Table Primers used for expression analyses in real time PCR analyses. (DOCX)

S3 Table Additional primers used in ChIP analyses. (DOCX)

S4 Table List of genes that are downregulated in HAT2-depleted cells. (XLSX)

S5 Table List of genes that are upregulated in HAT2-depleted cells. (XLSX)

S1 Fig **a. Tagging HAT2 genomic allele with eGFP**. Agarose gel electrophoresis analyses of PCRs across replacement junctions using genomic DNA as template. Positions of primers used are indicated in the line diagram and primer pairs used are indicated below the agarose gel images. Lanes 1: Ld1S, lanes 2: HAT2-eGFP tagged line, M: DNA ladder. ORCF-ORCR: PCR positive control. **b. IFA of HAT2-eGFP at different cell cycle stages**. DAPI: stains DNA compartments; N: nucleus, K: kinetoplast. G1/early S: one nucleus, one short kinetoplast (1N1K); late S/early G2/M: one nucleus, one elongated kinetoplast (1N1K); late G2/M: two nucleii, one kinetoplast (2N1K); post-mitosis—two nucleii, two kinetoplasts (2N2K).
(EPS)

S2 Fig Analysis of H4K10 acetylation. **a.** Western blot analysis of whole cell lysates isolated from promastigotes expressing HAT2-FLAG and HAT2-E332A-FLAG (4.5x10^7^ cell equivalents per lane) using anti-FLAG antibodies (1:5000 dilution; Sigma Aldrich). Ld1S-FLAG: cells carrying pXG-FLAG vector without HAT2 gene. 1/10 of each sample was loaded for tubulin control. **b.** Peptide Competition Assays. The specificity of the H4acetylK10 antibodies vis-à-vis being modification-specific as well as being specific to modification at the K10 residue of H4 was assessed as earlier [9]. Anti-H4acetylK10 antibodies were pre-incubated with various H4 peptides (8.5-fold or 85-fold in excess) prior to use in western blot analyses of *Leishmania* whole cell extracts. The H4acetylK10 antibodies did not cross-react with either unmodified H4 or H4acetylK4. **c.** Steady state levels of H4K10 acetylation were examined in logarithmically growing and stationary phase promastigotes, as well as in procyclic (non-infective form) and metacyclic (infective form) promastigotes (promastigotes: *Leishmania* stage in the insect host), using western blot analysis of whole cell lysates isolated from promastigotes at different stages (3x10^6^ cell equivalents per lane) using anti-H4K10 (1:1000 dilution), anti-H4K4 (1:1000 dilution), anti-H4 unmod (1:5000 dilution) antibodies (all custom-made by Abgent, USA), anti-tubulin (1:5000 dilution; Zymed). **d.** Examination of subcellular localization of H4K10 acetylation by immunofluorescence analysis at different cell cycle stages. DAPI: stains DNA compartment. N: nucleus, K: kinetoplast. G1/early S: one nucleus, one short kinetoplast (1N1K); late S/early G2/M: one nucleus, one elongated kinetoplast (1N1K); late G2/M: two nucleii, one kinetoplast (2N1K); post-mitosis—two nucleii, two kinetoplasts (2N2K).Magnification bar: 5 μm.
(EPS)

S3 Fig Analysis of HAT2 heterozygous knockout. **a & b.** Creation of HAT2-heterozygous knockout lines. **c.** Creation of HAT2-null in a HAT2^+^ background. Confirmation of all knockouts by PCRs across the deletion junctions, using primers designed against sequences within the donor cassettes in combination with primers designed against sequences lying in the *Leishmania* genome beyond the donor boundaries. Positions of primers used are indicated in the line diagram and primer pairs used are indicated below the agarose gel images. Lanes 1: Ld1S, lanes 2: HAT2-heterozygous knockout, M: DNA ladder. ORCF-ORCR: PCR positive control. **d.** Survival analyses of LdHAT2-hKO cells in comparison with control. Percent survivors was determined every 24 hours over a week. Three separate experiments were initiated in parallel. Values plotted are the average of three experiments, error bars represent standard deviation. Two-tailed student’s t-test was applied: *p < 0.05; **p < 0.005; ns:non-significant. **e.** Analysis of generation time. Growth was initiated from logarithmically growing cells, at 1x10^6^ cells/ml. Thereafter, cells were diluted back to 1x10^6^ cells/ml every 24 hours after counting. **f.** Western blot analysis of soluble and DNA-associated fractions of lysates isolated from Ld1S-hyg and LdHAT2-hKO:hyg cells (5x10^6^ promastigotes for each cell type). S1 and S2: soluble fractions, S3 and S4: DNA-associated fractions.
(EPS)

S4 Fig Genome map adapted from the *Leishmania donovani* genome map in the GeneDB (www.genedb.org; [45]). Genes that are downregulated in HAT2-depleted cells (based on microarray data) are depicted as green boxes and assigned an arabic numeral corresponding to their serial number in S4 Table. Predicted dSSRs and TSRs (transcription start regions) at chromosome ends are shaded light blue and ChIP-analyzed dSSRs are boxed; HT sites on chromosomes 5 and 35 shaded yellow; non-dSSR intergenic regions on chromosomes 5 and 32 that were analyzed in ChIPs are shaded pink. All analyzed S phase and mitotic cyclin genes (except CYC4 and CYC9), tubulin, HAT4, are depicted as red boxes. Analyzed genes coupled to chromosome 5 and chromosome 32 dSSRs as well as to chromosome 35 HT site are also depicted as red boxes. Gene clusters analyzed in run-ons are demarcated by red boxes.
(PDF)

S5 Fig Rescue of HAT2-hKO phenotype by coexpression of CYC4-eGFP and CYC9-FLAG. **a.** Western blot analysis of whole cell lysates isolated from LdHAT2-hKO cells expressing either CYC4-eGFP or CYC9-FLAG or coexpressing both, CYC4-eGFP and CYC9-FLAG. Lower panel: probed using anti-FLAG antibodies; upper panel: probed using anti-eGFP antibodies. **b.** Flow cytometry profiles of HU-synchronized LdHAT2-hKO cells co-expressing CYC4-eGFP and CYC9-FLAG. **c.** Analysis of expression of genes by real time PCR analysis in LdHAT2-hKO cells co-expressing CYC4-eGFP and CYC9-FLAG using 2^-ΔΔC^_T_ method (in which tubulin served as internal control).
(EPS)

S6 Fig Analysis of CYC4 and CYC9 upstream regions by ChIP. **a.** Left panel: Schematic representation depicting the 1 kb regions upstream of the start codon of the cyclin genes that were analyzed in reporter assays. SL sites: Spliced leader sites. All SL sites (indicated in red numbering with reference to downstream start codon which has been numbered “0”) have been obtained from www.tritrypdb.org (data provided to tritrypdb.org by Myler lab). All SL sites are with reference *Leishmania donovani* BPK282A1 except for CYC5 which is with reference to *Leishmania major* (SL site sequence conserved between *Leishmania donovani* and *Leishmania major*). Right panel: Map of pLEXSY vector used in reporter assays, taken from the Jena Bioscience website. **b.** IFA examining eGFP expression from CYC4 and CYC9 upstream regions in wild type and HAT2-depleted cells. **c.** ChIP analysis of logarithmically growing cells by real time PCR coupled to percent input method. Regions upstream of and within CYC4 and CYC9 genes were analyzed with “upstream primers” and “gene-specific primers.” Mock reactions without using antibodies–“Beads only”. **d.** Analysis of CYC4 and CYC9 upstream regions by ChIPs using H4acetylK10 antibodies, in HU-synchronized cells, at various time-points after release from block. Y-axis on a log_10_ scale. Three ChIP experiments were carried out, and in each of the three experiments real time PCR reactions were set up in triplicate. For each experiment the average value of each reaction was determined. Bar chart values presented here represent the mean of the three averages. Error bars indicate standard deviation. Two-tailed student’s t-test was applied: *p < 0.05, **p < 0.005, ***p < 0.0005. **e.** Flow cytometry profiles of synchronized cells whose RNA was isolated for analyses of CYC4 and CYC9 expression.
(EPS)

S7 Fig Shorter and longer exposures of nuclear run-ons. Left panels: schematic representations of slot blots indicating the blot position of each gene that was analyzed. At each time-point, slots corresponding to transcriptionally activated genes which matched with genes that were downregulated in HAT2-depleted cells are marked green while slots corresponding to transcriptionally activated genes that are linked to a dSSR are marked yellow. Other activated genes are marked mauve. Positive control: tubulin. Negative control: pUC19 plasmid with no insert. Centre and right panels: phosphorimaging of blots after hybridization with radiolabeled nascent RNA isolated from nuclei.
(PDF)

S8 Fig Schematic representation of dSSRs of chromosomes 5, 18 and 32, chromosome 35 HT region, and upstream regions of CYC4 and CYC9, showing locations of homopolymeric tracts. The lengths of the 5’UTRs were considered based on the SL addition site nearest to the start codon (based on data from the Myler lab available in TriTrypDB Kinetoplastid Genomics Resource (www.tritrypdb.org; [18]). Positions of homopolymeric tracts are marked with reference to the 5’ end of the considered 5’UTRs, which are marked as “0”.
(EPS)
