# Supplementary material for: Cell cycle stage-specific transcriptional activation of cyclins mediated by HAT2-dependent H4K10 acetylation of promoters in Leishmania donovani
Source: PLoS Pathog. 2017 Sep 22;13(9):e1006615. doi: 10.1371/journal.ppat.1006615 (PMC5627965; doi:10.1371/journal.ppat.1006615)
Supplement: S1 Methods — (DOCX) [file ppat.1006615.s001.docx]

**S1 Methods:**

***Leishmania* cultures**

*Leishmania* procyclics and metacyclics were isolated as described earlier [1]. Isolation of whole cell lysates, partitioning of whole cell lysates into soluble and DNA-associated fractions, and growth and survival analyses were carried out as previously detailed [2, 3]. Cell synchronization regimes with hydroxyurea and flavopiridol were carried out as earlier [3], and flow cytometry analyses were performed as described [4]. *Leishmania* transfections and creation of all clonal lines were done as previously [3].

To determine the generation time, mid-log phase cultures of Ld1S-hyg and HAT2-hKO:hyg were used to initiate cultures at 1x10^6^ cells/ml. Every 24 hours thereafter the cells were counted and cultures diluted to 1x10^6^ cells/ml with fresh M199. This was done for five days. Generation time was calculated as described [5] using the formula g = t / 3.3(log_10_N- log_10_N_0_) where t was the time interval (24 hours), N_0_ the initial cell count (1x10^6^ cells/ml) and N the cell count after 24 hours. The average generation time was determined from generation times calculated over five days.

***Cloning of HAT2 gene and expression in Leishmania for HAT assays***

The 1.8 kb *HAT2* gene amplicon was obtained using Phu DNA polymerase and *Leishmania donovani* 1S genomic DNA with the help of primers HAT2-GFP-F and HAT2-GFP-R (Table S1), and was cloned into pUC19 for sequencing. To express HAT2 in fusion with the FLAG tag in *Leishmania* for HAT assays, the gene was subcloned into the BamHI-EcoRV sites of pXG-/GFP+/FLAG [1], and the plasmid pXG/HAT2-FLAG so created was transfected into *Leishmania* promastigotes and clonal lines made as described earlier [3]. The LdHAT2-E332A mutant was created by overlap PCR using mutant primers HAT2-E332A-F and HAT2-E332A-R (Table S1) in combination with HAT2-GFP-R and HAT2-GFP-F primers respectively; followed by amplification of full-length gene using HAT2-GFP-F and HAT2-GFP-R. The full-length LdHAT2-E332A gene was cloned into pXG-/GFP+/FLAG, and the plasmid pXG/HAT2-E332A-FLAG so created transfected into *Leishmania* promastigotes, followed by screening of clonal lines for expression of the protein.

***Cloning of CYC4 and CYC9 genes and their expression in LdHAT2-hKO:hyg cells***

The CYC4 gene was cloned by amplification off genomic DNA using the primer pair CYC4-eGFP-F and CYC4-eGFP-R (Table S1), with the help of Phu DNA polymerase. The amplicon was directly cloned into the filled-in NcoI site of pLEXSY_I-egfp-neo3 (Jena Biosciences) such that LdCYC4 is correctly expressed in fusion with eGFP, creating plasmid pLEXSY-CYC4-eGFP. The CYC9 gene was cloned by amplification using primer pair CYC9-F and CYC9-R (Table S1), and inserting the amplicon into pUC19-SmaI site prior to resecting it with NcoI and cloning it into the NcoI site of pLEXSY_I-egfp-neo3 (such that CYC9 was expressed in fusion with FLAG tag at its C-terminus), thus creating plasmid pLEXSY-CYC9-FLAG. To create lines LdHAT2-hKO:hyg/CYC4-eGFP and LdHAT2-hKO:hyg/CYC9-FLAG, plasmids pLEXSY-CYC4-eGFP and pLEXSY-CYC9-FLAG were transfected into LdHAT2-hKO:hyg promastigotes respectively, and clones selected for using both drugs, hygromycin and G418.

To co-express CYC4-eGFP and CYC9-FLAG in *Leishmania* the CYC9-FLAG gene plus ~ 450 bp upstream sequence was resected from pLEXSY-CYC9-FLAG using EcoRI digestion (EcoRI upstream site in vector, downstream site in CYC9-R primer), and this cassette was cloned into the EcoRI site that lies ~ 450 bp upstream of the CYC4 gene in pLEXSY-CYC4-eGFP. The resultant plasmid pLEXSY-CYC4eGFP/CYC9-FLAG was transfected into LdHAT2-hKO:hyg promastigotes and clones selected for using G418.

***Cloning of upstream regions of cyclin genes***

The promoter upstream of the eGFP gene in pLEXSY_I-egfp-neo3 was deleted by EcoRI-BglII digestion of the vector followed by filling-in and religation, to create plasmid pLEXSY/egfpΔPr. The 1kb regions immediately upstream of the cyclin genes were amplified off genomic DNA using the following primer pairs: CYC4P-F and CYC4P-R to amplify the region upstream of the CYC4 gene, CYC5P-F and CYC5P-R to amplify the region upstream of the CYC5 gene, CYC8P-F and CYC8P-R to amplify the region upstream of the CYC8 gene, CYC9P-F and CYC9P-R to amplify the region upstream of the CYC9 gene, (primer sequences in Table S1). These amplicons were then used to replace the EcoRI-BglII fragment in pLEXSY_I-egfp-neo3. The plasmids created were named pLEXSY/egfpΔPr-CYC4P, pLEXSY/egfpΔPr-CYC5P, pLEXSY/egfpΔPr-CYC8P and pLEXSY/egfpΔPr-CYC9P.

***Raising modification-specific antibodies and analysis of their specificity by peptide competition assays***

Rabbits were immunized with H4acetylK10 and unmodified H4 peptides (two rabbits per peptide), and polyclonal antibodies to H4acetylK10 so obtained were affinity-purified against unmodified H4 peptide in order to obtain modification-specific antisera. The sequence of the peptide used for immunizations was AKGKRSADA**K**SSQKR (derived from the N-terminus of histone H4). The antibodies were raised and purified by Abgent, USA. The specificity of the antibodies to H4acetylK10 was checked using peptide competition assays as described [2]

***Tagging of HAT2 genomic allele with eGFP***

Tagging of one of the HAT2 genomic alleles with eGFP was done by homologous recombination using donor plasmid pLEXSY/HAT2-eGFP-neo/3’FL*.*  pLEXSY/HAT2-eGFP-neo was created by cloning the HAT2 gene (amplified off genomic DNA using primer pair HAT2-Rep-F and HAT2-Rep-R; Table S1) into the BglII site upstream of and in frame with the eGFP gene in pLEXSY-I-egfp-neo3. To create plasmid pLEXSY/HAT2-eGFP-neo/3’FL, the sequence downstream of the HAT2 gene (amplified off genomic DNA using primer pair HAT2-3’FL-F and HAT2-3’FL-R; Table S1) was inserted into the SpeI site downstream of the neomycin resistance cassette in pLEXSY/HAT2-eGFP-neo. The donor cassette for genomic allele replacement was released using EcoRI-StuI digestion (EcoRI site: in the vector just beyond the 5’ end of the inserted HAT2 gene; StuI site: at the end of the cloned 3’ Flank sequence in the amplification primer), gel purified, and transfected into Ld1S promastigotes. Clonals were selected for using G418 (50 μg/ml). The line generated was verified for genuine recombination at both ends using PCRs across the replacement junctions (Fig. S1b).

***Creation of HAT2 knockout and rescue lines***

Donor plasmids for creating genomic knockout lines were made using vectors pLEXSY_I-egfp-neo3 and pLEXSY-eGFP/hyg [3]. The 3’ Flank sequence of the HAT2 gene (same as above) was inserted into the SpeI site downstream of the neomycin/ hygromycin resistance cassette in pLEXSY_I-egfp-neo3 and pLEXSY-eGFP/hyg respectively. This was followed by insertion of the sequence upstream of the HAT2 gene (amplified off genomic DNA using primer pair HAT2-5’Fl-F and HAT2-5’FL-R; Table S1) into the NotI site downstream of the eGFP gene in both constructs, thus creating knockout plasmids pHAT2-KO/neo and pHAT2-KO/hyg*.* Donor cassettes were released from pHAT2-KO-neo and pHAT2-KO-hyg using StuI digestion (one site 160 bp into the 5’Flank sequence, and second site at the end of the cloned 3’ Flank sequence in the amplification primer), gel purified, and transfected into Ld1S promastigotes. Clonals of heterozygous knockout lines LdHAT2-hKO:neo and LdHAT2-hKO:hyg were selected for using G418 (50 μg/ml) and hygromycin (16 μg/ml) respectively. To create heterozygous knockout rescue line, plasmid pLEXSY/HAT2-eGFP-neo was transfected into LdHAT2-hKO:hyg promastigotes. Clones of LdHAT2-hKO:hyg/HAT2-eGFP (rescue line) were selected for using G418 and hygromycin and screened for robust HAT2-eGFP expression by western blotting.

To create HAT2-null line, LdHAT2-hKO:hyg cells were transfected with donor cassette released from pHAT2-KO/neo plasmid and transfection mix plated for selection of clones using hygromycin and G418. To create HAT2-null line using LdHAT2-hKO:hyg cells expressing HAT2 episomally as the background line, LdHAT2-hKO:hyg promastigotes were first transfected with pLEXSY/HAT2-eGFP-blecherry (created by replacing the *neo* resistance cassette in pLEXSY/HAT2-eGFP-neo with the *blecherry* cassette from pLEXSY_blecherry (Jena Biosciences) with the help of BamHI-SpeI digestion), and a clone expressing HAT2-eGFP robustly was used. These LdHAT2-hKO:hyg/HAT2-eGFP-blecherry promastigotes were now transfected with the donor cassette released from pHAT2-KO/neo, and transfection mix plated for selection of clones using hygromycin, G418, and bleomycin (2.5 μg/ml).

***Immunofluorescence analysis***

Immunofluorescence experiments for examining the subcellular localization of HAT2-eGFP and H4acetylK10 were carried out as detailed previously [2]. Anti-eGFP (mouse) antibodies were used at 1:100 dilution; anti-H4unmodified (rabbit) antibodies and anti-H4acetylK10 (rabbit) antibodies were used at 1:1000 dilution and 1:500 dilution respectively; anti-mouse secondary antibodies labeled with FITC and anti-rabbit secondary antibodies labeled with Texas Red (both from Jackson Immunoresearch Laboratories, USA) were used at 1:200 dilution. After processing, the cells were mounted in anti-fade solution containing DAPI (Vectashield, Vector Laboratories). Images were captured with a LeicaTCS SP5 confocal microscope using a 100X (in oil) objective at room temperature, and analyzed using Leica LAS AF software.

**References:**

1. Minocha N, Kumar D, Rajanala K, Saha S. Characterization of Leishmania donovani MCM4: expression patterns and interaction with PCNA. PLoS One. 2011;6(7):e23107. Epub 2011/08/11. doi: 10.1371/journal.pone.0023107

PONE-D-11-08097 [pii]. PubMed PMID: 21829589.

2. Kumar D, Saha S. HAT3-mediated acetylation of PCNA precedes PCNA monoubiquitination following exposure to UV radiation in Leishmania donovani. Nucleic Acids Res. 2015;43(11):5423-41. doi: 10.1093/nar/gkv431. PubMed PMID: 25948582; PubMed Central PMCID: PMC4477661.

3. Yadav A, Chandra U, Saha S. Histone acetyltransferase HAT4 modulates navigation across G2/M and re-entry into G1 in Leishmania donovani. Sci Rep. 2016;6:27510. doi: 10.1038/srep27510. PubMed PMID: 27272906; PubMed Central PMCID: PMCPMC4897741.

4. Minocha N, Kumar D, Rajanala K, Saha S. Kinetoplast morphology and segregation pattern as a marker for cell cycle progression in Leishmania donovani. J Eukaryot Microbiol. 2011;58(3):249-53. Epub 2011/03/16. doi: 10.1111/j.1550-7408.2011.00539.x. PubMed PMID: 21401783.

5. Pelczar MJ, Chan, E.C.S. Microbiology (An Application Based Approach): Tata McGraw-Hill; 2010.

**Legends for Supporting Tables:**

**S1 Table:** List of primers used for clonings

**S2 Table:** Primers used for expression analyses in real time PCR analyses

**S3 Table:** Additional primers used in ChIP analyses

**S4 Table:** List of genes that are downregulated in HAT2-depleted cells

**S5 Table:** List of genes that are upregulated in HAT2-depleted cells

**Legends for Supporting Figures:**

**S1 Fig: a.** **Tagging HAT2 genomic allele with eGFP**. Agarose gel electrophoresis analyses of PCRs across replacement junctions using genomic DNA as template. Positions of primers used are indicated in the line diagram and primer pairs used are indicated below the agarose gel images. Lanes 1: Ld1S, lanes 2: HAT2-eGFP tagged line, M: DNA ladder. ORCF-ORCR: PCR positive control**. b. IFA of HAT2-eGFP at different cell cycle stages**. DAPI: stains DNA compartments; N: nucleus, K: kinetoplast. G1/early S: one nucleus, one short kinetoplast (1N1K); late S/early G2/M: one nucleus, one elongated kinetoplast (1N1K); late G2/M: two nucleii, one kinetoplast (2N1K); post-mitosis - two nucleii, two kinetoplasts (2N2K).

**S2 Fig: Analysis of H4K10 acetylation a.** Western blot analysis of whole cell lysates isolated from promastigotes expressing HAT2-FLAG and HAT2-E332A-FLAG (4.5x10^7^ cell equivalents per lane) using anti-FLAG antibodies (1:5000 dilution; Sigma Aldrich). Ld1S-FLAG: cells carrying pXG-FLAG vector without HAT2 gene. 1/10 of each sample was loaded for tubulin control. **b.** Peptide Competition Assays. The specificity of the H4acetylK10 antibodies vis-à-vis being modification-specific as well as being specific to modification at the K10 residue of H4 was assessed as earlier [2]. Anti-H4acetylK10 antibodies were pre-incubated with various H4 peptides (8.5-fold or 85-fold in excess) prior to use in western blot analyses of *Leishmania* whole cell extracts. The H4acetylK10 antibodies did not cross-react with either unmodified H4 or H4acetylK4. **c.** Steady state levels of H4K10 acetylation were examined in logarithmically growing and stationary phase promastigotes, as well as in procyclic (non-infective form) and metacyclic (infective form) promastigotes (promastigotes: *Leishmania* stage in the insect host), using western blot analysis of whole cell lysates isolated from promastigotes at different stages (3x10^6^ cell equivalents per lane) using anti-H4K10 (1:1000 dilution), anti-H4K4 (1:1000 dilution), anti-H4 unmod (1:5000 dilution) antibodies (all custom-made by Abgent, USA), anti-tubulin (1:5000 dilution; Zymed). **d.** Examination of subcellular localization of H4K10 acetylation by immunofluorescence analysis at different cell cycle stages. DAPI: stains DNA compartment. N: nucleus, K: kinetoplast. G1/early S: one nucleus, one short kinetoplast (1N1K); late S/early G2/M: one nucleus, one elongated kinetoplast (1N1K); late G2/M: two nucleii, one kinetoplast (2N1K); post-mitosis - two nucleii, two kinetoplasts (2N2K).Magnification bar: 5 μm.

**S3 Fig: Analysis of HAT2 heterozygous knockout a & b.** Creation of HAT2-heterozygous knockout lines. **c.** Creation of HAT2-null in HAT2^+^ background. Confirmation of all knockouts by PCRs across the deletion junctions, using primers designed against sequences within the donor cassettes in combination with primers designed against sequences lying in the *Leishmania* genome beyond the donor boundaries. Positions of primers used are indicated in the line diagram and primer pairs used are indicated below the agarose gel images. Lanes 1: Ld1S, lanes 2: HAT2-heterozygous knockout, M: DNA ladder. ORCF-ORCR: PCR positive control. **c.** Survival analyses of LdHAT2-hKO cells in comparison with control. Percent survivors was determined every 24 hours over a week. Three separate experiments were initiated in parallel. Values plotted are the average of three experiments, error bars represent standard deviation. Two-tailed student’s t-test was applied: **p* < 0.05; ***p* < 0.005; ns:non-significant. **d.** Analysis of generation time. Growth was initiated from logarithmically growing cells, at 1x10^6^ cells/ml. Thereafter, cells were diluted back to 1x10^6^ cells/ml every 24 hours after counting. **e.** Western blot analysis of soluble and DNA-associated fractions of lysates isolated from Ld1S-hyg and LdHAT2-hKO:hyg cells (5x10^6^ promastigotes for each cell type). S1 and S2: soluble fractions, S3 and S4: DNA-associated fractions.

**S4 Fig: Genomic maps adapted from the *Leishmania donovani* genome maps in the GeneDB (www.genedb.org)**. Genes that are downregulated in HAT2-depleted cells (based on microarray data) are depicted as green boxes and assigned an arabic numeral corresponding to their serial number in S4 Table. Predicted dSSRs and TSRs (transcription start regions) at chromosome ends are shaded light blue and ChIP-analyzed dSSRs are boxed; HT sites on chromosomes 5 and 35 shaded yellow; non-dSSR intergenic regions on chromosomes 5 and 32 that were analyzed in ChIPs are shaded pink. All analyzed S phase and mitotic cyclin genes (except CYC4 and CYC9), tubulin, HAT4, are depicted as red boxes. Analyzed genes coupled to chromosome 5 and chromosome 32 dSSRs as well as to chromosome 35 HT site are also depicted as red boxes. Gene clusters analyzed in run-ons are demarcated by red boxes.

**S5 Fig: Rescue of HAT2-hKO phenotype by coexpression of CYC4-eGFP and CYC9-FLAG a.** Western blot analysis of whole cell lysates isolated from LdHAT2-hKO cells expressing either CYC4-eGFP or CYC9-FLAG or coexpressing both, CYC4-eGFP and CYC9-FLAG. Lower panel: probed using anti-FLAG antibodies; upper panel: probed using anti-eGFP antibodies. **b.** Flow cytometry profiles of HU-synchronized LdHAT2-hKO cells co-expressing CYC4-eGFP and CYC9-FLAG. **c.** Analysis of expression of genes by real time PCR analysis in LdHAT2-hKO cells co-expressing CYC4-eGFP and CYC9-FLAG using 2^-ΔΔC^_T_ method (in which tubulin served as internal control).

**S6 Fig: Analysis of CYC4 and CYC9 upstream regions by ChIP. a.** Left panel: Schematic representation depicting the 1 kb regions upstream of the start codon of the cyclin genes that were analyzed in reporter assays. SL sites: Spliced leader sites. All SL sites (indicated in red numbering with reference to downstream start codon which has been numbered “0”) have been obtained from [www.tritrypdb.org](http://www.tritryp.org) (data provided to tritrypdb.org by Myler lab). All SL sites are with reference *Leishmania donovani* BPK282A1 except for CYC5 which is with reference to *Leishmania major* (SL site sequence conserved between *Leishmania donovani* and *Leishmania major*). Right panel: Map of pLEXSY vector used in reporter assays, taken from the Jena Bioscience website. **b.** IFA examining eGFP expression from CYC4 and CYC9 upstream regions in wild type and HAT2-depleted cells. **c.** ChIP analysis of logarithmically growing cells by real time PCR coupled to percent input method. Regions upstream of and within CYC4 and CYC9 genes were analyzed with “upstream primers” and “gene-specific primers.” Mock reactions without using antibodies – “Beads only”. **d.** Analysis of CYC4 and CYC9 upstream regions by ChIPs using H4acetylK10 antibodies, in HU-synchronized cells, at various time-points after release from block. Y-axis on a log_10_ scale. Three ChIP experiments were carried out, and in each of the three experiments real time PCR reactions were set up in triplicate. For each experiment the average value of each reaction was determined. Bar chart values presented here represent the mean of the three averages. Error bars indicate standard deviation. Two-tailed student’s t-test was applied: **p* < 0.05, ***p* < 0.005, ****p* < 0.0005. **e.** Flow cytometry profiles of synchronized cells whose RNA was isolated for analyses of CYC4 and CYC9 expression.

**S7 Fig: Shorter and longer exposures of nuclear run-ons**. Left panels: schematic representations of slot blots indicating the blot position of each gene that was analyzed. At each time-point, slots corresponding to transcriptionally activated genes which matched with genes that were downregulated in HAT2-depleted cells are marked green while slots corresponding to transcriptionally activated genes that are linked to a dSSR are marked yellow. Other activated genes are marked mauve. Positive control: tubulin. Negative control: pUC19 plasmid with no insert. Centre and right panels: phosphorimaging of blots after hybridization with radiolabeled nascent RNA isolated from nuclei.

**S8 Fig: Schematic representation of dSSRs of chromosomes 5, 18 and 32, chromosome 35 HT region, and upstream regions of CYC4 and CYC9, showing locations of homopolymeric tracts**. The lengths of the 5’UTRs were considered based on the SL addition site nearest to the start codon (based on data from the Myler lab available in TriTrypDB Kinetoplastid Genomics Resource (www.tritrypdb.org). Positions of homopolymeric tracts are marked with reference to the 5’ end of the considered 5’UTRs, which are marked as “0”.
