# Supplementary material for: Cell cycle stage-specific transcriptional activation of cyclins mediated by HAT2-dependent H4K10 acetylation of promoters in Leishmania donovani
Source: PLoS Pathog. 2017 Sep 22;13(9):e1006615. doi: 10.1371/journal.ppat.1006615 (PMC5627965; doi:10.1371/journal.ppat.1006615)
Supplement: S1 Table — (DOCX) [file ppat.1006615.s002.docx]

**Table S1:** Primers used for clonings

| Primer name | Primer sequence |
| --- | --- |
| HAT2-GFP-F | 5’-CACCGGATCCACCATGGCTGTCGCGCA-3’ |
| HAT2-GFP-R | 5’-TCCGATATCTCCGCGTGTGCTGG-3’ |
| HAT2-E332A-F | 5’-GGCACCCCGGCGAAGCCCCTCTCCGACTTG-3’ |
| HAT2-E332A-R | 5’- CAAGTCGGAGAGGGGCTTCGCCGGGGTGCC-3’ |
| CYC4-eGFP-F | 5’- CACCCCATGGCCACCATGGACTGTGTAGAA-3’ |
| CYC4-eGFP-R | 5’- ACCATGGTCCGTAGTCCTTGTAGCT-3’ |
| CYC9-F | 5’- ATCCATGGCACCATGTCGTCAAGCCCAT-3’ |
| CYC9-R | 5’-TACCATGGGAATTCTCACTTGTCATCGTCGTCCTTGTAGTC  AGATCTTCCCTGCTCCGACATCTT-3’ |
| CYC4P-F | 5’-ATGAATTCCACCCTTCTCGCCCA 3’ |
| CYC4P-R | 5’-ATGGATCCGAAAGGAAGGGTACGG 3’ |
| CYC5P-F | 5’-ATGAATTCGGTATCTCGGCGAGCGT 3’ |
| CYC5P-R | 5’-ATGGATCCCGGATCATCCCCCGAA 3’ |
| CYC8P-F | 5’-ATGAATTCCTGACGCTGATGGAGCT 3’ |
| CYC8P-R | 5’-ATGGATCCGAGAGGAGCGGCATGA 3’ |
| CYC9P-F | 5’-ATGAATTCGACGTGCATGGGCCA 3’ |
| CYC9P-R | 5’-ATGGATCCATTGCGGTGTAACGCA 3’ |
| HAT2-Rep-F | 5’- TCAGATCTATGGCTGTCGCGCA-3’ |
| HAT2-Rep-R | 5’- TGAGATCTTCCGCGTGTGCTGG-3’ |
| HAT2-3’FL-F | 5’-TCACTAGTCACTTTCTCTCTCTCGTTCCT-3’ |
| HAT2-3’FL-R | 5’-TAACTAGTAGGCCTGTTGTACTTTGCTCAAGCGCT-3’ |
| HAT2-5’Fl-F | 5’-TAGCGGCCGCCCAGTTAGTGTCACCTCGCT-3’ |
| HAT2-5’FL-R | 5’-TCGCGGCCGCTACGTAGACGTCGTCTGCAT-3’ |
