# Supplementary material for: Cell cycle stage-specific transcriptional activation of cyclins mediated by HAT2-dependent H4K10 acetylation of promoters in Leishmania donovani
Source: PLoS Pathog. 2017 Sep 22;13(9):e1006615. doi: 10.1371/journal.ppat.1006615 (PMC5627965; doi:10.1371/journal.ppat.1006615)
Supplement: S2 Table — (DOCX) [file ppat.1006615.s003.docx]

**Table S2:** Primers used for expression analyses in real time PCR analyses:

| TriTrypDB  accession number  and gene name | Primer name | Primer sequence |
| --- | --- | --- |
| LdBPK_282440.1  HAT2 | HAT2-RT-F | 5’- TGACGTGCTCAGGACGCT-3’ |
|  | HAT2-RT-R | 5’-TGAGAACCGCGGGACTTG-3’ |
| LdBPK_320870.1  CYC2 | CYC2-RT-F | 5’-CTACGCCAGCATTGGGGGCGTGGT-3’ |
|  | CYC2-RT-R | 5’-GAAGAGTGCCTCTAGCCGAGCTACGTA-3’ |
| LdBPK_300080.1  CYC3 | CYC3-RT-F | 5’-CTCGCGCTGTGTCTTGT-3’ |
|  | CYC3-RT-R | 5’- TACTTGGAGGTAAGCAGGT-3’ |
| LdBPK_050710.1  CYC4 | CYC4-RT-F | 5’-CACCCCCACACCTTCCCAG-3’ |
|  | CYC4-RT-R | 5’-GCCGAGTGAGTGCTGCGA-3’ |
| LdBPK_330830.1  CYC5 | CYC5-RT-F | 5’-ACTCCCACGACGAGGAT-3’ |
|  | CYC5-RT-R | 5’-CCTCGTACCACGCGGT-3’ |
| LdBPK_323520.1  CYC6 | CYC6-RT-F | 5’-GAGGTCATCCTAGATTATGT-3’ |
|  | CYC6-RT-R | 5’- ATCCTAGATTATGTGAACTA-3’ |
| LdBPK_303690.1  CYC7 | CYC7-RT-F | 5’-ATTACCATGTCGTCATGGTGGA-3’ |
|  | CYC7-RT-R | 5’-AGCCAAGATCTCCTGGGGTCGA-3’ |
| LdBPK_260320.1  CYC8 | CYC8-RT-F | 5’-GCGAGGCACTTCTGCTCCA-3’ |
|  | CYC8-RT-R | 5’-AGTACGCGCACGGGCTCA-3’ |
| LdBPK_320800.1  CYC9 | CYC9-RT-F | 5’-ACACCGCTCTGCATCAAGGTG-3’ |
|  | CYC9-RT-R | 5’-ACTTCTTGTGCAGGTACG-3’ |
| LdBPK_130330.1  Tubulin | Tub-RT-F1 | 5’-CTTCAAGTGCGGCATCAACTA-3’ |
|  | Tub-RT-R2 | 5’-TTAGTACTCCTCGACGTCCTC-3’ |
| LdBPK_140440.1  mRNA cap. enz. | Cap. enz-RT-F | 5’-TCGGCGCTTGTGGATACAAC-3’ |
|  | Cap. enz-RT-R | 5’-TTCGGCCACGCGCAAGAGA-3’ |
| LdBPK_323910.1  Enolase | Enolase-RT-F | 5’-TCTTCAGTGCGCACACAGCT-3’ |
|  | Enolase-RT-R | 5’-TCACCCTTCCGGCGGATC-3’ |
| LdBPK_151040.1  Rad9 | Rad9-RT-F | 5’-GCTGCCTTCCGCGTCACTG-3’ |
|  | Rad9-RT-R | 5’-CTCGCGCAGCTCAGCATCC-3’ |
| LdBPK_332880.1  IF2 | IF2-RT-F | 5’-ACCCCTGGCATGGACCTGG-3’ |
|  | IF2-RT-R | 5’-CATCCGGCTTCAGCTCGTC-3’ |
| LdBPK_050900.1  Surface Ag-like protein | Ag-RT-F | 5’-GCTGCCTCCCCTCGGAGT-3’ |
|  | Ag-RT-R | 5’-ACGCTCGCGCGGCTGCAG-3’ |
| LdBPK_050140.1  Stomatin-like protein | Stomatin-RT-F | 5’-GCGAAGCACAGCAACGAGG-3’ |
|  | Stomatin-RT-R | 5’-TGAACACGGAGAGTGCTTGG-3’ |
| LdBPK_050150.1  Hypothetical protein | HP1-RT-F | 5’-GGCATCAAGCTTTTCACGGA-3’ |
|  | HP1-RT-R | 5’-GGCGCGCAGCTTTGAGG-3’ |
| LdBPK_320510.1  Hypothetical protein | HP2-RT-F | 5’-GACGGATTTCCCACGCGG-3’ |
|  | HP2-RT-R | 5’-CTTCGAGGCGCTCTATAATAC-3’ |
| LdBPK_320520.1  Rab4 | Rab4-RT-F | 5’-GCAGTCCAGCACAACGAGGCAT-3’ |
|  | Rab4-RT-R | 5’-AGCTGGGTGTCGTTTTCTGG-3’ |
| LdBPK_351210.1  RNA helicase | RNAh-RT-F | 5’-GTATTTAACGAGCTAGTCCTC-3’ |
|  | RNAh-RT-R | 5’-CCTTGCCTTCGGAAAATCTC-3’ |
| LdBPK_351220.1  Arginine-rich protein | Arg-RT-F | 5’-CTGGCTCTCACCTCTTTCAG-3’ |
|  | Arg-RT-R | 5’-CAATCCCGACATTGGTAGTCC-3’ |
