# Supplementary material for: Cell cycle stage-specific transcriptional activation of cyclins mediated by HAT2-dependent H4K10 acetylation of promoters in Leishmania donovani
Source: PLoS Pathog. 2017 Sep 22;13(9):e1006615. doi: 10.1371/journal.ppat.1006615 (PMC5627965; doi:10.1371/journal.ppat.1006615)
Supplement: S3 Table — (DOCX) [file ppat.1006615.s004.docx]

**Table S3:** Additional primers used in ChIP analyses:

| Gene whose upstream  region is analysed | Primer name | Primer sequence |
| --- | --- | --- |
| CYC2 | CYC2-UP/188Dn | 5’- CACCGCCGACAGCGATGGACT-3’ |
|  | CYC2-UP/10Up | 5’- GCAACATCTACTGGGGCCGCAAT-3’ |
| CYC3 | Mit.cyc2-UP/213Dn | 5’- AAACGTGTTCCCTACGGTCGAGA -3’ |
|  | Mit.cyc2-UP/9Up | 5’- ATGCCCACACGTCGGTGGATGA -3’ |
| CYC4 | CYC4-UP/189Dn | 5’- TCATCTGCCTCCTCGGTTGCTT-3’ |
|  | CYC4-UP/10Up | 5’- GTACGGCGGACACGCAC-3’ |
| CYC5 | CYC5-UP/189Dn | 5’- ATACCGATCCAAGGGACGGA-3’ |
|  | CYC5-UP/12Up | 5’- CGAAGTACCGCCGACGAGCGA-3’ |
| CYC6 | CYC6-UP/174Dn | 5’- TCCATGCACAGTTTGGTGGT-3’ |
|  | CYC6-UP/8Up | 5’- TGTCGCCAATCAAATCCGT-3’ |
| CYC7 | CYC7-UP/201Dn | 5’- AGACTTAAACGCGGTACAAAGA-3’ |
|  | CYC7-UP/10Up | 5’- AACACAATGGTTTCCCTGTCA-3’ |
| CYC8 | Mit.cyc1-UP/212Dn | 5’- TCTTTTCCACACTTGCACAGCGAT-3’ |
|  | Mit.cyc1-UP/12Up | 5’- TCTCTTGGAGATGGTTTACCGGT-3’ |
| CYC9 | CYC9-UP/186Dn | 5’- TCCGTTGTCGAGCTGGACGT-3’ |
|  | CYC9-UP/9Up | 5’- TAACGCAGGGTCCTTTGTGAT-3’ |
| Tubulin | Tub-UP/205Dn | 5’-GAAGAAGAGGGGTTAGAAGG-3’ |
|  | Tub-UP/11Up | 5’-CCATGTGCCGGACGTCTG-3’ |
| HAT4 | HAT4-UP/202Dn | 5’-TGTGCGTGCAGGTGTGTCA-3’ |
|  | HAT4-UP/12Up | 5’-CACCCCTGCGCTGCAGG-3’ |
| Chromosome 5 dSSR  (384450-385900) | Ch5/384460Dn | 5’- ACTCGCATTTGATTCTTGAGTTTA-3’ |
|  | Ch5/384620Up | 5’- AAGACGAGCGTTGAACAGAAGAC-3’ |
| Chromosome 32 dSSR  (181745-183630) | Ch32/183481Dn | 5’- TTGTATGCACCATGCTGCCAGT-3’ |
|  | Ch32/183609Up | 5’- TAAAGCCAACACAAACAGACACGT-3’ |
| Chromosome 5  IG region  (379160-378210) | Ch5/378401Dn | 5’- CTCTCAAGGCCTCGCGCA-3’ |
|  | Ch5/378219Up | 5’-TCAACACCTCGCGCTCTCT-3’ |
| Chromosome 32  IG region  (176140-178699) | Ch32/176328Dn | 5’- CAGTGTCTCAGCAACTCAGG |
|  | Ch32/176144Up | 5’- TATCGAGACAGACAGCCACAC-3’ |
| Chromosome 35  HT region  (534085-538756) | Ch35-HT/538564Dn | 5’-AGCCACGTAATCGAACAGTAC-3’ |
|  | Ch35-HT/538748Up | 5’-ACCTACTCTCTTTCCATCTTGA-3’ |
