# Supplementary material for: Cell cycle stage-specific transcriptional activation of cyclins mediated by HAT2-dependent H4K10 acetylation of promoters in Leishmania donovani
Source: PLoS Pathog. 2017 Sep 22;13(9):e1006615. doi: 10.1371/journal.ppat.1006615 (PMC5627965; doi:10.1371/journal.ppat.1006615)
Supplement: S4 Fig — Genes that are downregulated in HAT2-depleted cells (based on microarray data) are depicted as green boxes and assigned an arabic numeral corresponding to their serial number in S4 Table. Predicted dSSRs and TSRs (transcription start regions) at chromosome ends are shaded light blue and ChIP-analyzed dSSRs are boxed; HT sites on chromosomes 5 and 35 shaded yellow; non-dSSR intergenic regions on chromosomes 5 and 32 that were analyzed in ChIPs are shaded pink. All analyzed S phase and mitotic cyclin genes (except CYC4 and CYC9), tubulin, HAT4, are depicted as red boxes. Analyzed genes coupled to chromosome 5 and chromosome 32 dSSRs as well as to chromosome 35 HT site are also depicted as red boxes. Gene clusters analyzed in run-ons are demarcated by red boxes. (PDF) [file ppat.1006615.s010.pdf]

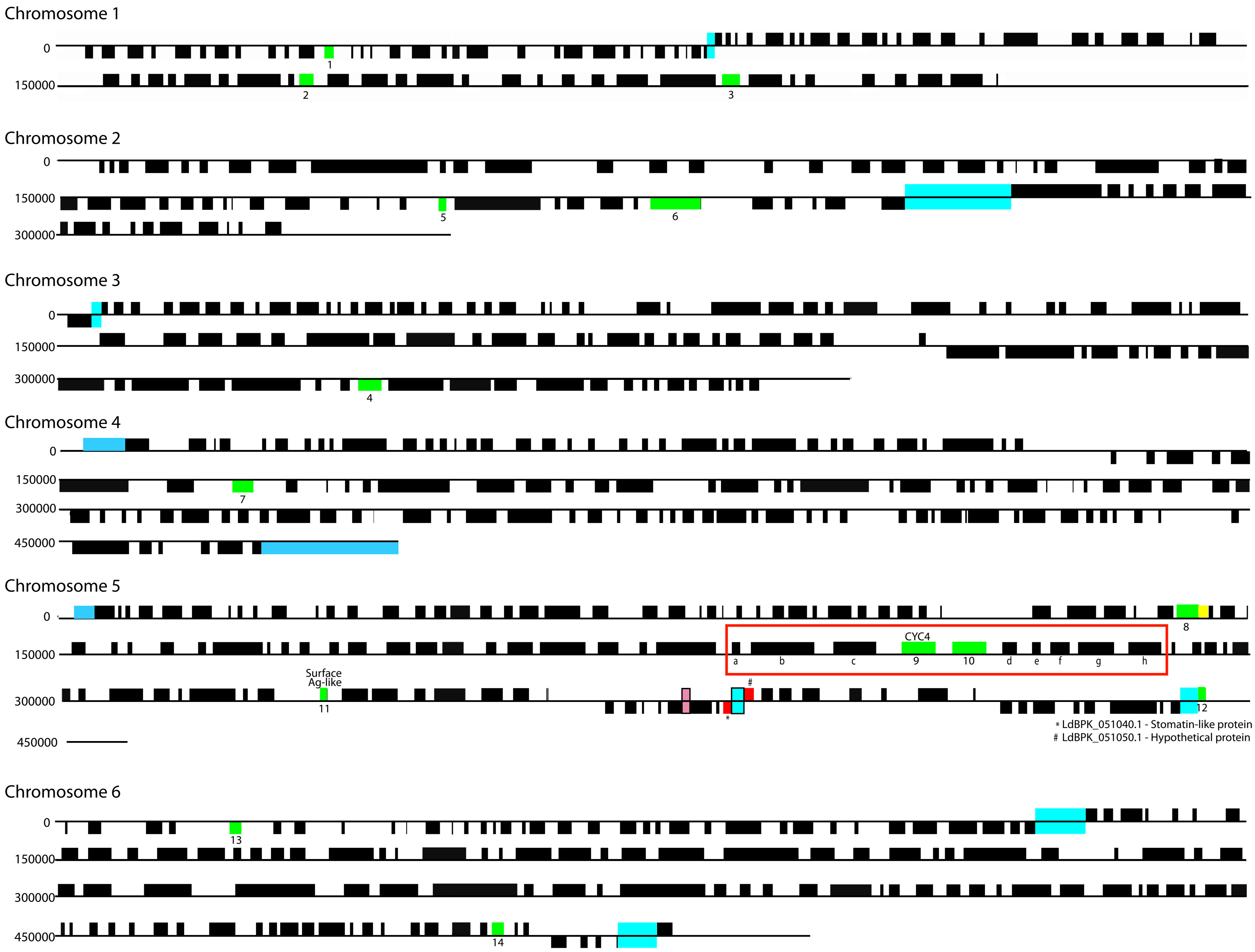

Chromosome 1
0
150000
1
2
3
Chromosome 2
0
150000
300000
5
6
Chromosome 3
0
150000
300000
4
Chromosome 4
0
150000
300000
450000
7
Chromosome 5
0
150000
300000
450000
8
CYC4
a
b
c
9
10
d
e
f
g
h
Surface
Ag-like
11
#
*
12
* LdBPK_051040.1 - Stomatin-like protein
# LdBPK_051050.1 - Hypothetical protein
Chromosome 6
0
150000
300000
450000
13
14

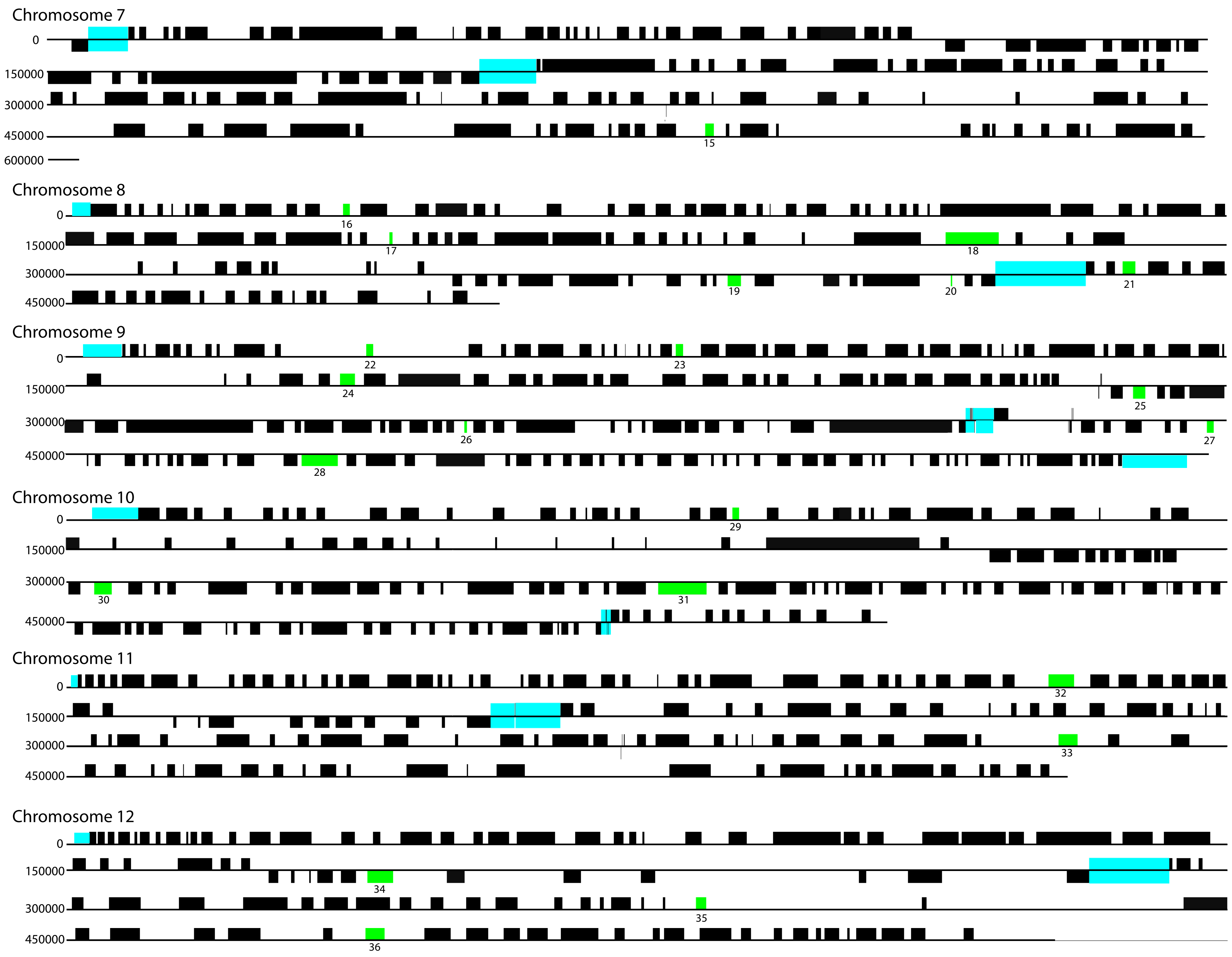

Chromosome 7
0
150000
300000
450000
600000
15
Chromosome 8
0
150000
300000
450000
16
17
18
19
20
21
Chromosome 9
0
150000
300000
450000
22
23
24
25
26
27
28
Chromosome 10
0
150000
300000
450000
29
30
31
Chromosome 11
0
150000
300000
450000
32
33
Chromosome 12
0
150000
300000
450000
34
35
36

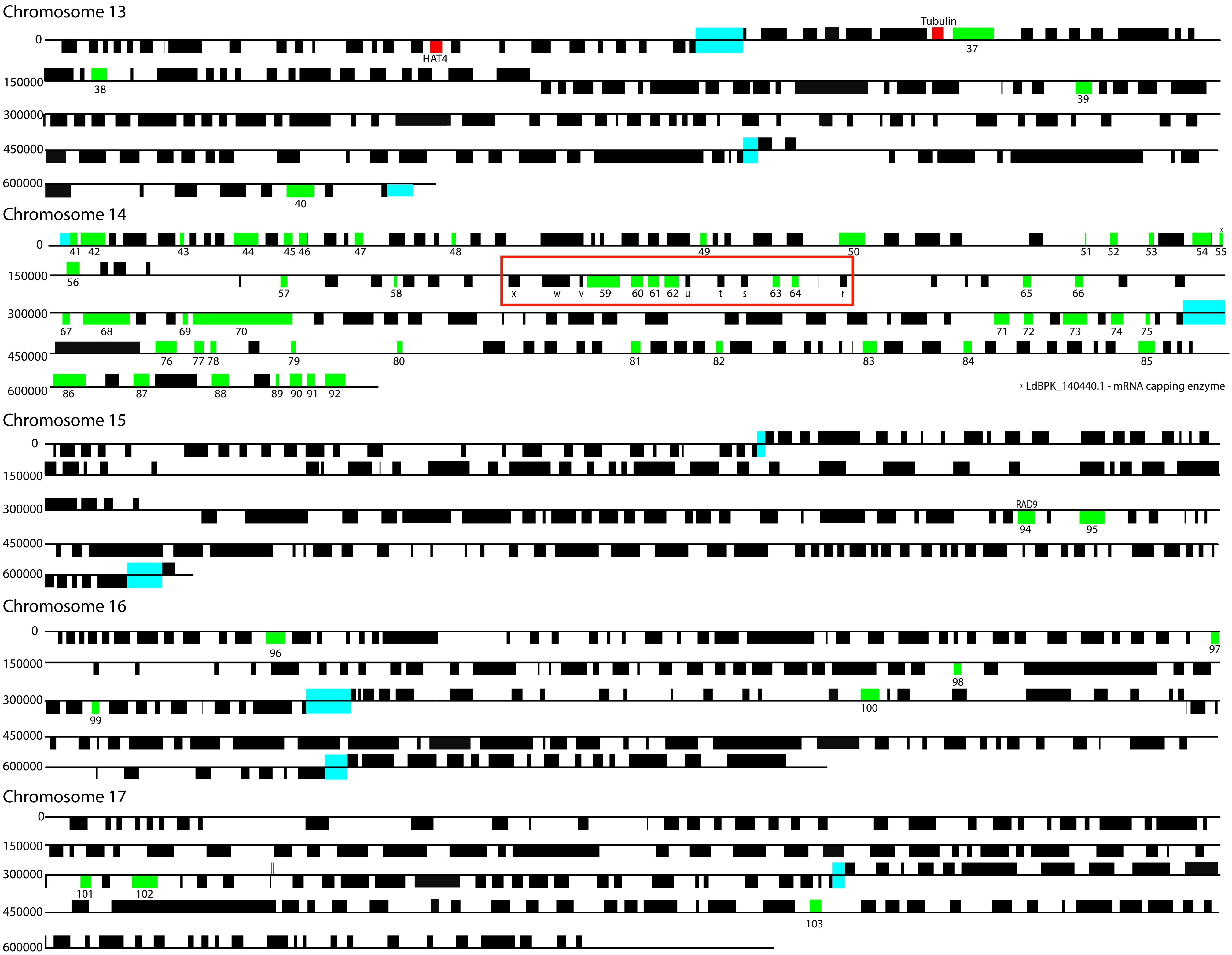
Chromosome 13
0
150000
300000
450000
600000
Tubulin
HAT4
37
38
39
40
Chromosome 14
0
150000
300000
450000
600000
*
41 42 43 44 45 46 47 48 49 50 51 52 53 54 55
56
x w v 59 60 61 62 u t s 63 64 r
57 58 65 66
67 68 69 70 71 72 73 74 75
76 77 78 79 80 81 82 83 84 85
86 87 88 89 90 91 92
* LdBPK_140440.1 - mRNA capping enzyme
Chromosome 15
0
150000
300000
450000
600000
RAD9
94 95
Chromosome 16
0
150000
300000
450000
600000
96 97 98 99 100
Chromosome 17
0
150000
300000
450000
600000
101 102 103

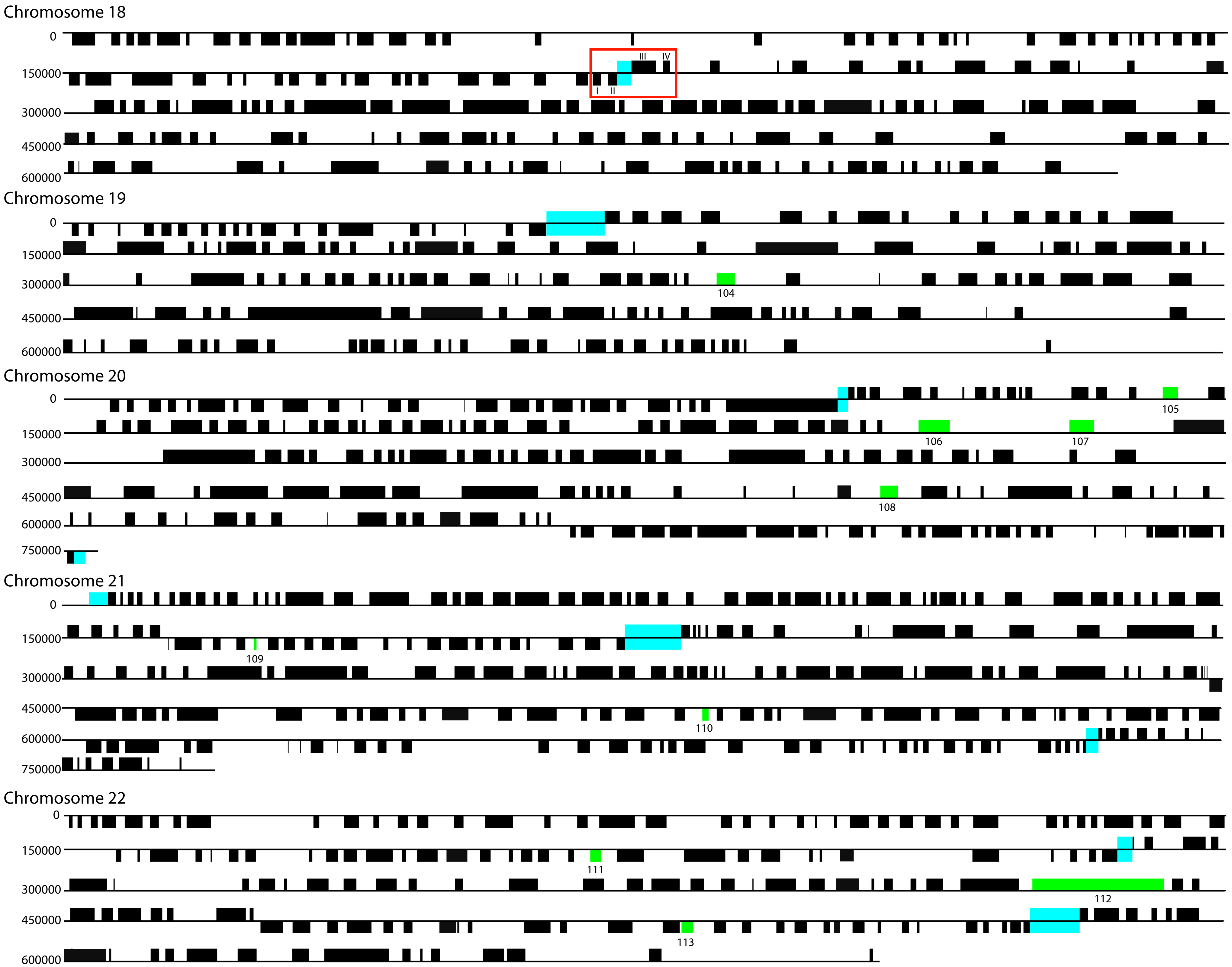
Chromosome 18
0
150000
300000
450000
600000
I
II
III
IV
Chromosome 19
0
150000
300000
450000
600000
104
Chromosome 20
0
150000
300000
450000
600000
750000
105
106
107
108
Chromosome 21
0
150000
300000
450000
600000
750000
109
110
Chromosome 22
0
150000
300000
450000
600000
111
112
113

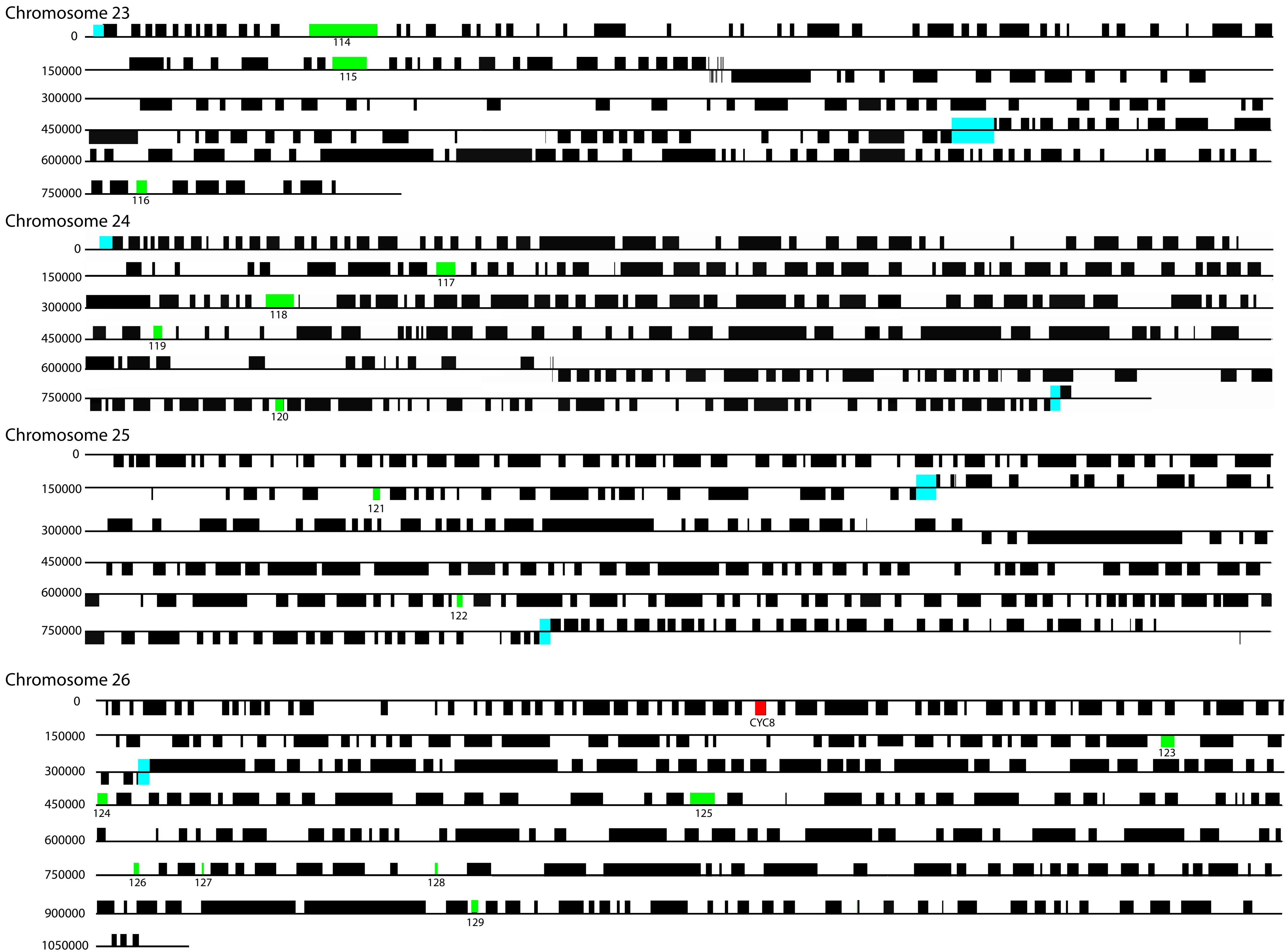

Chromosome 23
0
114
150000
115
300000
450000
600000
750000
116
Chromosome 24
0
150000
117
300000
118
450000
119
600000
750000
120
Chromosome 25
0
150000
121
300000
450000
600000
122
750000
Chromosome 26
0
CYC8
150000
123
300000
450000
124
125
600000
750000
126
127
128
900000
129
1050000

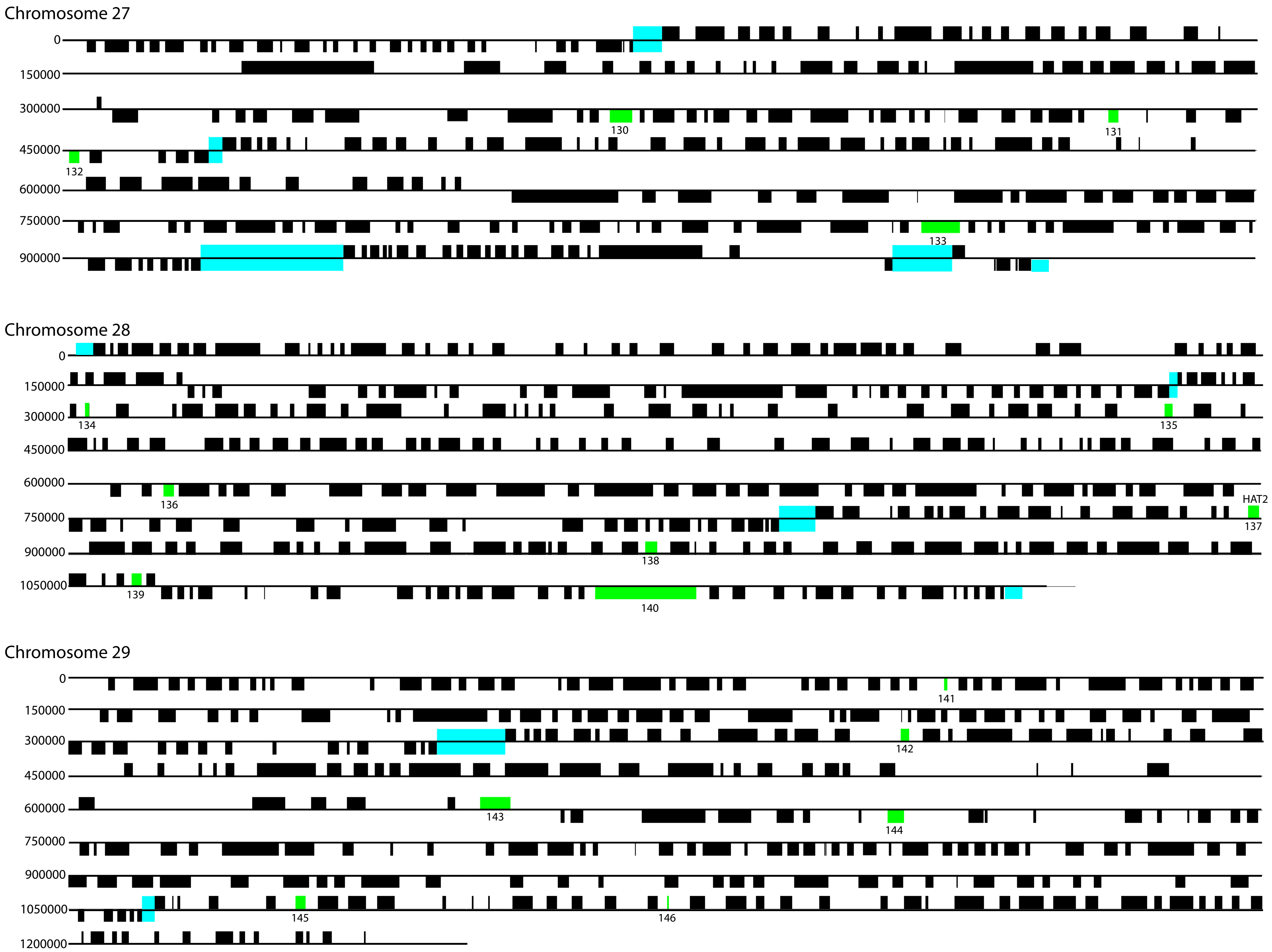
Chromosome 27
0
150000
300000
450000
600000
750000
900000
130
131
132
133
Chromosome 28
0
150000
300000
450000
600000
750000
900000
1050000
134
135
136
HAT2
137
138
139
140
Chromosome 29
0
150000
300000
450000
600000
750000
900000
1050000
1200000
141
142
143
144
145
146

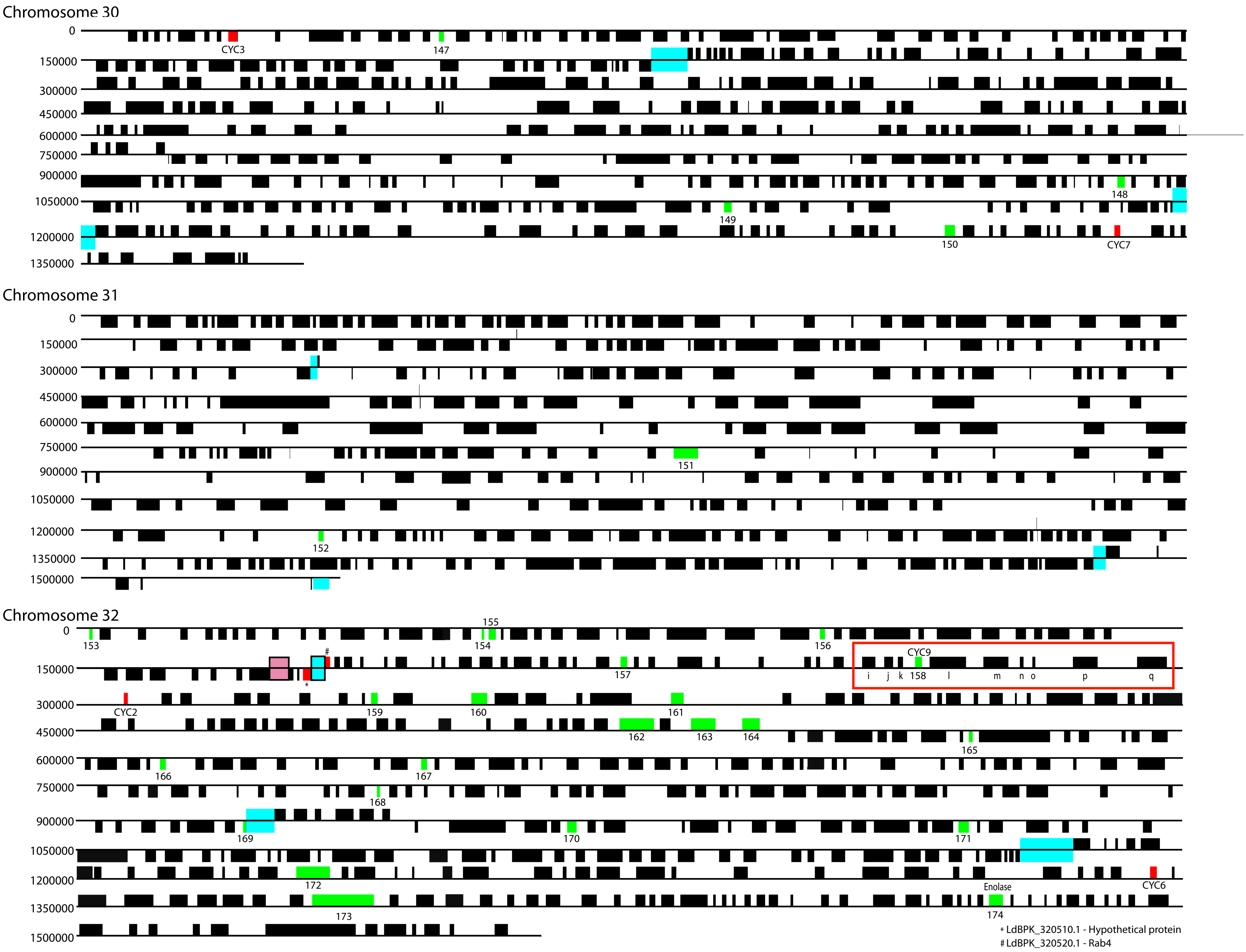
Chromosome 30
0
150000
300000
450000
600000
750000
900000
1050000
1200000
1350000
CYC3
147
148
149
150
CYC7
Chromosome 31
0
150000
300000
450000
600000
750000
900000
1050000
1200000
1350000
1500000
151
152
Chromosome 32
0
150000
300000
450000
600000
750000
900000
1050000
1200000
1350000
1500000
153
154
155
156
#
*
157
CYC9
i j k 158 l m n o p q
CYC2
159
160
161
162
163
164
165
166
167
168
169
170
171
172
Enolase
CYC6
173
174
* LdBPK_320510.1 - Hypothetical protein
# LdBPK_320520.1 - Rab4

Chromosome 33

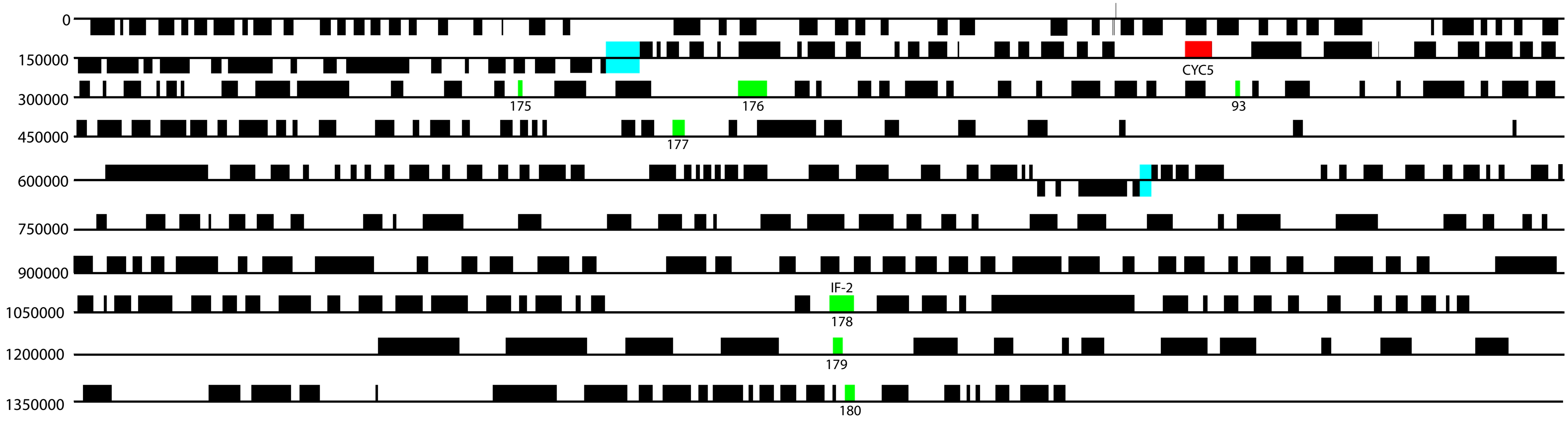

Chromosome 34

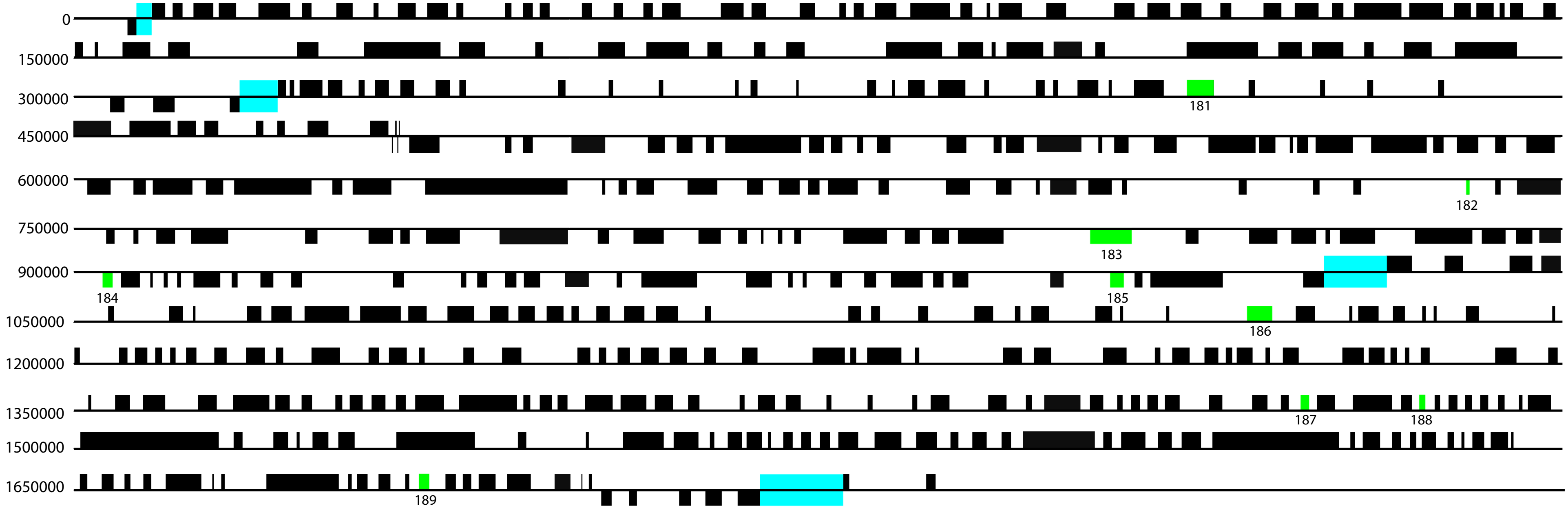

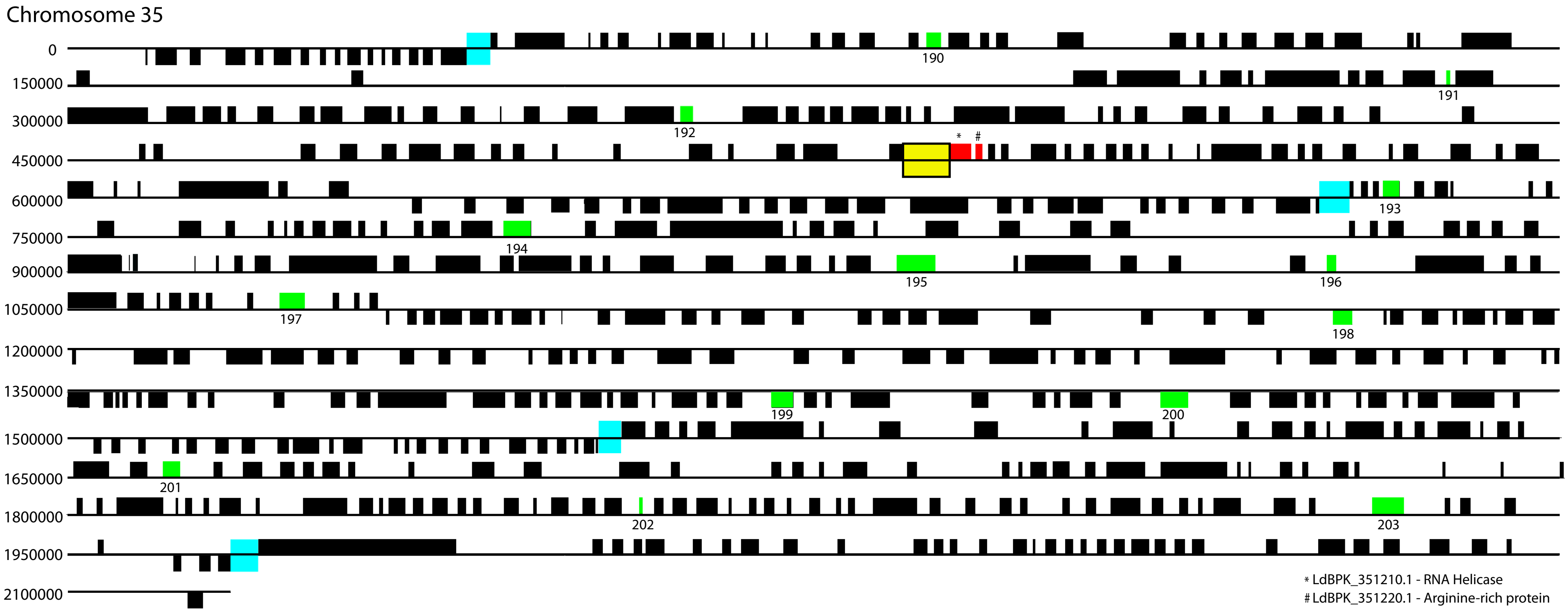
Chromosome 35
0
150000
300000
450000
600000
750000
900000
1050000
1200000
1350000
1500000
1650000
1800000
1950000
2100000
190
191
192
*
#
193
194
195
196
197
198
199
200
201
202
203
* LdBPK_351210.1 - RNA Helicase
# LdBPK_351220.1 - Arginine-rich protein

Chromosome 36

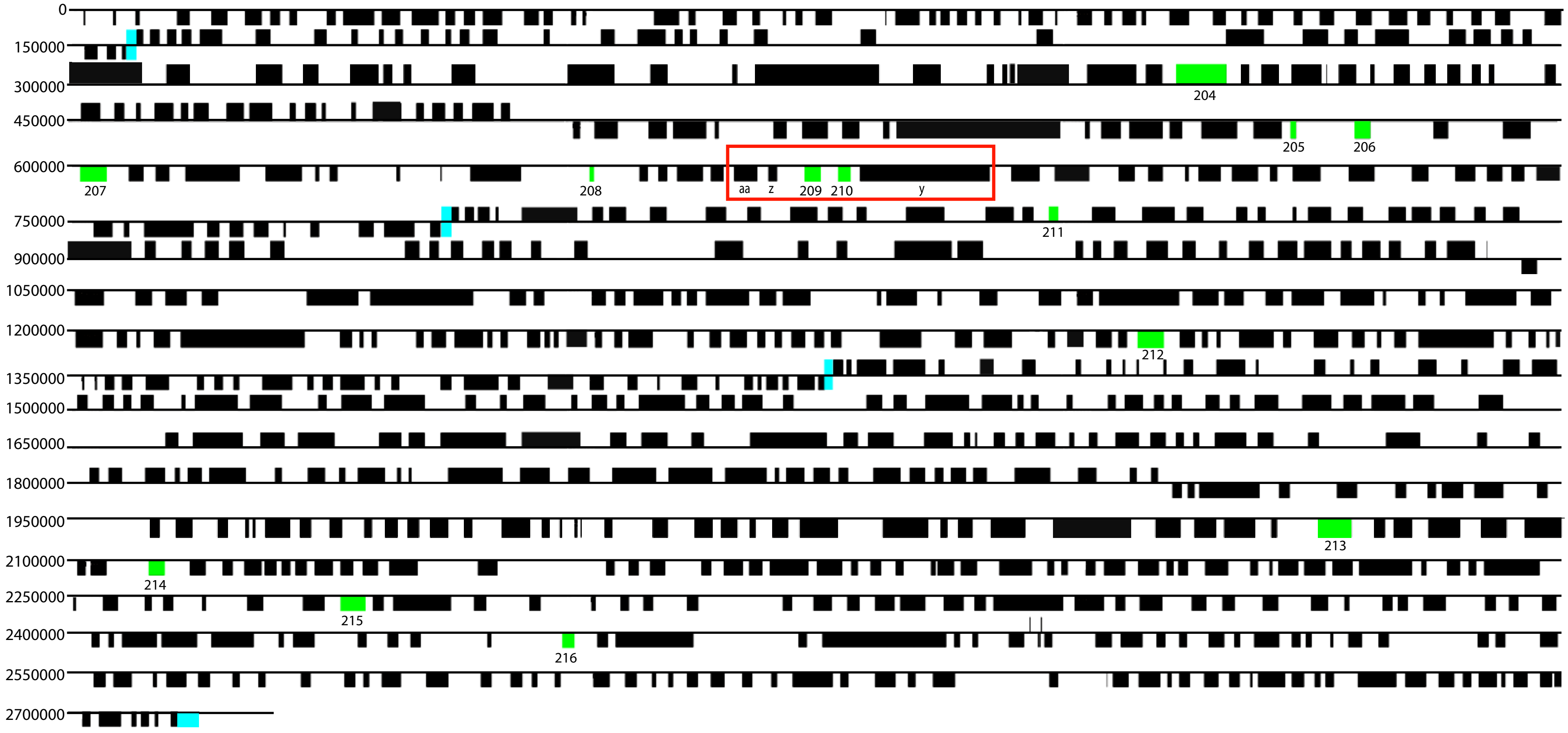

S4 Fig: Genome map adapted from the *Leishmania donovani* genome map in the GeneDB (www.genedb.org). Genes that are downregulated in HAT2-depleted cells (based on microarray data) are depicted as green boxes and assigned an arabic numeral corresponding to their serial number in S4 Table. Predicted dSSRs and TSRs (transcription start sites) at chromosome ends are shaded light blue and ChIP-analyzed dSSRs are boxed; HT sites on chromosomes 5 and 35 shaded yellow; non-dSSR intergenic regions on chromosomes 5 and 32 that were analyzed in ChIPs are shaded pink. All analyzed S phase and mitotic cyclin genes (except CYC4 and CYC9), tubulin, HAT4, are depicted as red boxes. Analyzed genes coupled to chromosome 5 and chromosome 32 dSSRs as well as to chromosome 35 HT site are also depicted as red boxes. Gene clusters analyzed in run-ons are demarcated by red boxes.
