# Supplementary material for: Cell cycle stage-specific transcriptional activation of cyclins mediated by HAT2-dependent H4K10 acetylation of promoters in Leishmania donovani
Source: PLoS Pathog. 2017 Sep 22;13(9):e1006615. doi: 10.1371/journal.ppat.1006615 (PMC5627965; doi:10.1371/journal.ppat.1006615)
Supplement: S7 Fig — Left panels: schematic representations of slot blots indicating the blot position of each gene that was analyzed. At each time-point, slots corresponding to transcriptionally activated genes which matched with genes that were downregulated in HAT2-depleted cells are marked green while slots corresponding to transcriptionally activated genes that are linked to a dSSR are marked yellow. Other activated genes are marked mauve. Positive control: tubulin. Negative control: pUC19 plasmid with no insert. Centre and right panels: phosphorimaging of blots after hybridization with radiolabeled nascent RNA isolated from nuclei. (PDF) [file ppat.1006615.s013.pdf]

S7 Figure

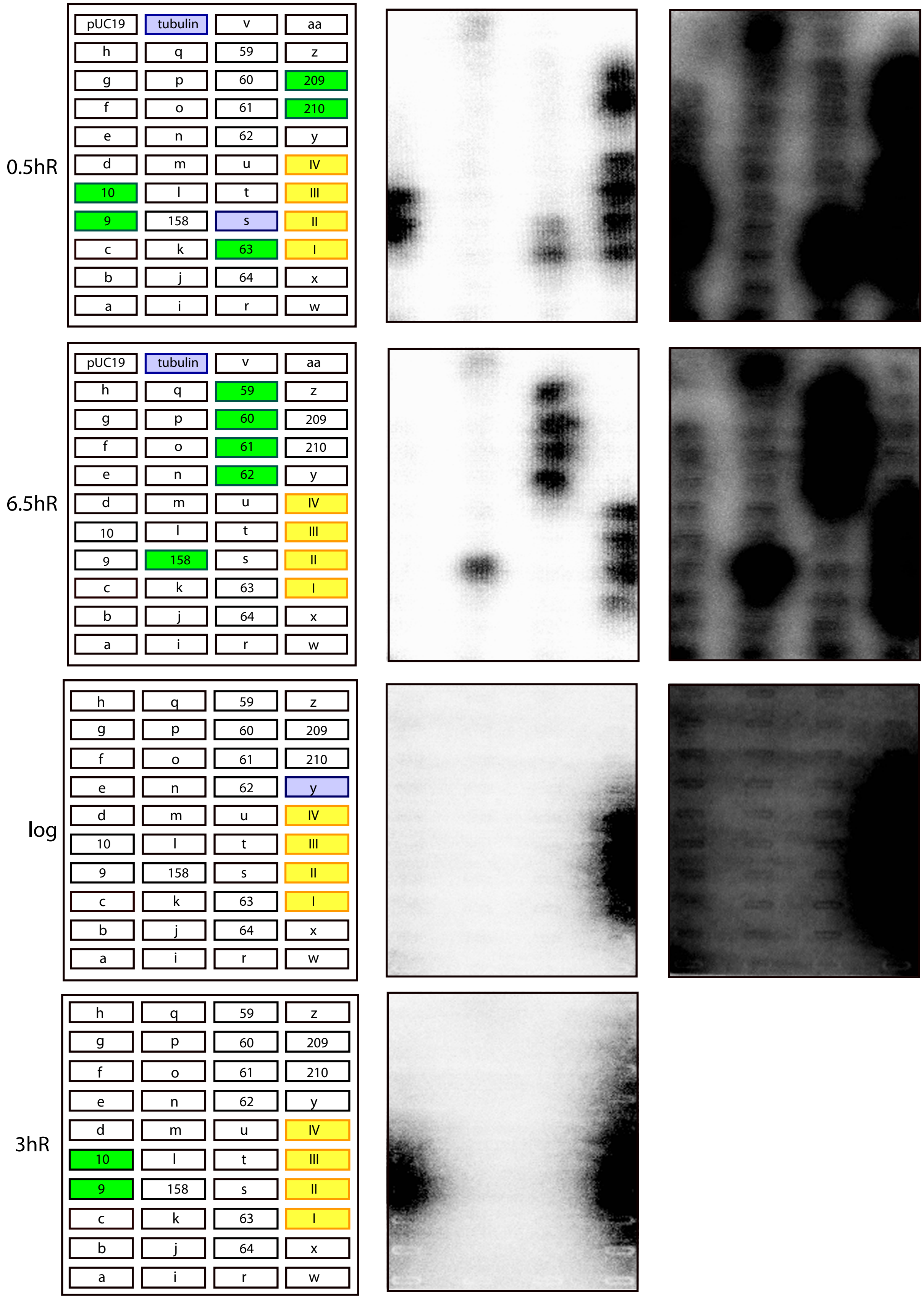

S7 Fig: Shorter and longer exposures of nuclear run-ons. Left panels: schematic representations of slot blots indicating the blot position of each gene that was analyzed. At each time-point, slots corresponding to transcriptionally activated genes which matched with genes that were downregulated in HAT2-depleted cells are marked green while slots corresponding to transcriptionally activated genes that are linked to a dSSR are marked yellow. Other activated genes are marked mauve. Positive control: tubulin. Negative control: pUC19 plasmid with no insert. Centre and right panels: phosphorimaging of blots after hybridization with radiolabeled nascent RNA isolated from nuclei.
